# Fertilization influences the substrate, rhizosphere, and endosphere bacteriome of *Petunia × hybrida*

**DOI:** 10.3389/fmicb.2025.1719754

**Published:** 2025-12-05

**Authors:** Juan Quijia-Pillajo, Laura J. Chapin, James S. Owen, James E. Altland, Michelle L. Jones

**Affiliations:** 1 Department of Horticulture and Crop Science, The Ohio State University, Wooster, OH, United States; 2 Application Technology Research Unit, United States Department of Agriculture (USDA)-Agricultural Research Service, Wooster, OH, United States

**Keywords:** microbiome, ammonium, peat, floriculture, controlled environment agriculture, 16S rRNA, amplicon sequencing

## Abstract

**Introduction:**

In controlled environment agriculture (CEA), soil is replaced with soilless substrates that have poorly understood microbiome dynamics. We investigated the rhizosphere and endosphere bacteriome of *Petunia* × *hybrida* ‘Picobella Blue’ (Picobella) and ‘Wave Purple’ (Wave) grown in a soilless substrate (80% sphagnum peat and 20% perlite) under three fertilization rates (25, 100, and 200 mg·L^−1^ N).

**Methods:**

Plant growth was assessed with the TraitFinder phenotyping platform, shoot dry weight, and nutrient analysis. Bacteriomes were profiled by 16S rRNA amplicon sequencing from unplanted substrate, bulk substrate, rhizosphere, and endosphere samples.

**Results:**

Both cultivars grew largest and healthiest at 200 mg·L^−1^ N. Picobella fertilized with 100 and 200 mg·L^−1^ N were equally green, whereas Wave was greenest at 200 mg·L^−1^ N. Distinct bacteriomes were observed across unplanted substrate, rhizosphere, and endosphere. In unplanted substrate, fertilizer rate shaped bacterial community composition but not alpha diversity. In the rhizosphere, pH changes driven by fertilization strongly influenced bacterial community structure and reduced diversity. Endosphere and rhizosphere communities were further shaped by cultivar and fertilization rate.

**Discussion:**

These findings highlight nutrient management as a key driver of bacteriome dynamics across the substrate–plant continuum, underscoring the complex interactions between fertilization, plant genotype, and microbial communities in soilless culture.

## 1 Introduction

The plant microbiome, consisting of all microbial communities living on or inside the plant organs, plays a fundamental role in plant development and health (Compant et al., 2025). Soil serves as the primary source of microbial diversity (Banerjee and van der Heijden, 2023). Below ground, microbiome assembly is described by a two-step selection process. First, microbes are selectively recruited from the soil to the rhizosphere (2–4 mm of soil closely attached to the root system), and subsequently, a subset of these microbes colonize the root endosphere (Bulgarelli et al., 2013). Microbe recruitment is driven by the release of root exudates into the rhizosphere, where microbial activity is prolific (Reinhold-Hurek et al., 2015). Colonization of the root endosphere is modulated by the plant immune system (Bulgarelli et al., 2013; Reinhold-Hurek et al., 2015; Trivedi et al., 2020).

Soil microbial communities are modulated by its physical and chemical properties (soil pH, nutrient availability, soil moisture, and temperature) (Custódio et al., 2022). The soil as a microbial source greatly determines rhizosphere and endosphere microbial communities and function (Lundberg et al., 2012). As important as soil is, the host plant is another strong determinant of the root microbiome (Fitzpatrick et al., 2018). The host-plant effect on microbiome assembly is explained by differences in the root exudate composition between plant species. Moreover, the “cry-for-help” hypothesis states that the root exudate profile can be further modulated in response to biotic and environmental stress to recruit specific microbiota that contribute to stress tolerance (Pascale et al., 2020). The plant microbiome is considered an extension of the plant genome since plant associated microbes contribute capabilities that the plant host lacks and thereby increase plant host fitness (Vandenkoornhuyse et al., 2015).

A hallmark of controlled environment agriculture (CEA) systems is the replacement of soil by soilless substrate mixes for growing crops in containers. In such systems, the substrate, irrigation water, and microbial inoculants are key microbial sources for root microbiome assembly (Montagne et al., 2017; Picot et al., 2020; Tan et al., 2021; Valles-Ramirez et al., 2023). Soilless substrates are mixes of organic (i.e., peat, coir, wood fiber, and bark) and inorganic (i.e., perlite and vermiculite) material optimized to maximize plant performance (Carlile et al., 2019). Despite environmental concerns regarding peat extraction, peat remains the most widely used organic substrate component. The popularity of peat lies in properties like high total porosity resulting in high water-holding capacity and adequate air space, low pH, and high cation exchange capacity (Nau et al., 2021). While the physical (i.e., particle size and air porosity) and chemical (i.e., pH buffering capacity and fertilizer charge) properties of substrates are well studied (Silber, 2019; Wallach, 2019), their microbiological characteristics remain less understood.

Recent studies have shown that microbial community composition in bulk soilless substrates is shaped by several factors, including the source material, physical structure, and manufacturing processes (Montagne et al., 2017; Pot et al., 2022; Valles-Ramirez et al., 2023). The pH is a known key factor driving soil microbial community structure (Custódio et al., 2022). Consequently, changes in pH (e.g., by liming or ammonium fertilization) influence the microbial composition of soil and soilless substrates (Yin et al., 2021; Pot et al., 2022). Heavy fertilization is common in container-grown crops using soilless substrates, and the ammonium to nitrate (NH_4_^+^:NO_3_^−^) ratio in the fertilizer solution influences growth and substrate pH (Nau et al., 2021). In soil, the rhizosphere bacterial diversity and community composition depends on pH changes associated with NH_4_^+^ and NO_3_^−^ uptake by the root (Zhang et al., 2021). While it is well established that fertilization influences soil microbiomes, its effects on soilless substrate microbiomes are less well understood (Kong et al., 2023; Quijia Pillajo et al., 2024). For instance, Quijia Pillajo et al. (2024) showed that increasing fertilization rate changed bacterial community composition in a peat-based substrate, and that those changes were driven by substrate pH and phosphate concentration. Due to the diversity of cultivated ornamental crop genera, species, and cultivars, varying fertilizer formulations, and individualized substrate mixes available, these findings highlight the need for a deeper understanding of how plant species, substrate properties, and cultural management practices shape microbial communities in soilless culture.

Although peat has been considered an ineffective medium for harboring beneficial microorganisms (Hoitink and Boehm, 1999; Krause et al., 2001; Pot et al., 2022), both commercially available and novel inoculants are reported to promote growth or reduce abiotic stress in petunia and other ornamentals grown in peat-based soilless substrates (Nordstedt and Jones, 2021; South et al., 2021; Lin and Jones, 2022). For instance, *Caballeronia zhejiangensis* C7B12 promoted growth in petunia ‘Picobella Blue’ grown under limited fertilization (25 mg·L^−1^ N) (South et al., 2021), and *Serratia plymuthica* MBSA-MJ1 promoted growth and reduced yellowing in petunia ‘Picobella Blue’ and *Impatiens walleriana* ‘Super Elfin Ruby’ exposed to drought stress (Nordstedt and Jones, 2021).

*Petunia × hybrida* is a popular greenhouse ornamental and model plant system that requires high fertilization (150–250 mg·L^−1^ N) and is susceptible to iron deficiency at basic pH (Gibson et al., 2007; Nau et al., 2021). Petunia crop production research has primarily focused on optimizing fertilizer regimes and substrate pH, with more recent efforts exploring the use of bacterial inoculants (Park and Faust, 2021; South et al., 2021). However, little attention has been given to the naturally associated microbial communities of petunia. To our knowledge only a single study has reported that the petunia (line V26) root microbiome is modulated in response to phosphorus fertilization in soil. Under low-P conditions, the petunia microbiome was enriched by microorganisms reported to support P nutrition (arbuscular mycorrhizal fungi and bacteria of the order Burkholderiales and Rhodocyclales) (Bodenhausen et al., 2019).

The efficacy of microbial inoculants depends on interactions with native microbiomes (Ferrarezi et al., 2023). Thus, advancement of microbial inoculants in CEA requires better understanding of existing microbiomes and factors influencing them. The value of microorganisms in supporting plant growth and health is widely acknowledged in conventional agriculture (Compant et al., 2025). Similarly, there is an increasing amount of research showing the potential of microbial inoculants for soilless culture systems. However, while soil microbiome research has provided valuable insights for the application of microbial inoculants in soilless culture, the microbiome of substrates used in CEA remains relatively underexplored. This project contributes to global efforts to better understand the substrate microbiome and develop strategies to advance soilless cultivation. The objectives of this study were (1) to describe the rhizosphere and endosphere bacteriome of petunia grown in a peat-based substrate, and (2) to evaluate how fertilization rates and different hybrid cultivars of the same genus modulate microbiome assembly.

## 2 Materials and methods

### 2.1 Plant material and greenhouse culture

For clarity, *Petunia × hybrida* ‘Picobella Blue’ (Syngenta Flowers, Gilroy, CA, USA) and ‘Wave Purple’ (PanAmerican Seed, West Chicago, IL, USA) here after are referred to as Picobella and Wave, respectively throughout the text. Petunia seeds were sown in 288-size plug trays filled with a peat-vermiculite germination substrate (Pro-Mix PGX, Premier Tech Horticulture, Quakertown, PA, USA). Plug trays were covered with a plastic dome to maintain humidity and germinated under fluorescent lights at room temperature (~ 21 °C). The seedlings were fertilized 14 and 21 days after sowing. The fertilizer solution was prepared from the water-soluble fertilizer Jack’s Professional 20N–1.3P–15.8K petunia FeED (J.R. Peters, Allentown, PA, USA) at a rate of 25 mg N per liter of solution (mg·L^−1^ N). In this formulation, N was provided as 60% ammonium (NH_4_^+^) and 40% nitrate (NO_3_^−^). Twenty-seven days after sowing, seedlings were transplanted into 15.2-cm round pots filled with a peat-based, soilless substrate and watered with the corresponding fertilizer treatment solution. The soilless substrate contained 80% sphagnum peat (Pro-Moss, Premier Tech Horticulture, Quakertown, PA, USA) and 20% coarse perlite (PVP Industries Inc., North Bloomfield, OH, USA) by volume. The substrate was amended with pulverized dolomitic limestone (calcium carbonate equivalent = 95%; Oldcastle Lawn & Garden, Atlanta, GA, USA) to achieve a final pH of 5.5. Wetting agent (Aqua- GrowL, Aquatrols, Paulsboro, NJ, USA) was supplemented at 7.7 mL per 100 L of substrate. Plants were grown in a controlled environment greenhouse with a 14 h photoperiod. Supplemental light was provided by a 1:1 mix of metal halide and high-pressure sodium lamps when the photosynthetically active radiation (PAR) was less than 250 μmol·m^−2^·s^−1^ at canopy level. Temperature at canopy level was set to 21–24 °C during the day and 16–18 °C during the night. The greenhouse relative humidity was set to 70%, and shade was provided when the PAR was higher than 400 μmol·m^−2^·s^−1^.

Pots were organized in a randomized complete block design (RCBD) with a 3 × 3 factorial arrangement and seven blocks (*n* = 7). Treatments were the combination of petunia cultivar (Unplanted substrate, Picobella, and Wave) and fertilizer level (25, 100, and 200 mg·L^−1^ N). Unplanted pots were included in the experimental design to test the effect of fertilizer alone on the substrate chemical properties and bacteriome in absence of the plant. The fertilizer treatments were prepared using the same water-soluble fertilizer previously described. Three solutions with low (25 mg·L^−1^ N), medium (100 mg·L^−1^ N), and high (200 mg·L^−1^ N) fertilizer levels were used in this experiment. The experiment was terminated 7 weeks after transplant.

### 2.2 Plant evaluation

At the end of the experiment, digital biomass, canopy hue, green leaf index (GLI), normalized difference vegetation index (NDVI), normalized pigment chlorophyll index (NPCI), plant senescence reflectance index (PSRI), and proportion of green and yellow canopy were measured using a digital phenotyping platform (TraitFinder; Phenospex, Heerlen, The Netherlands). To avoid any influence of flower tissue on GLI, NDVI, NPCI, and PSRI, the data was collected before and after manually removing flowers (Quijia Pillajo et al., 2023). Canopy hue was used to calculate the proportion of green (125°–180°) and yellow (30°–90°) coloration in the canopy (Quijia-Pillajo et al., 2025). After digital phenotyping, open flowers, buds, and shoots were removed and placed in paper bags. The collected plant tissue was dried in a forced-air oven at 60 °C for at least 7 days. Dry weights were recorded, and shoot samples were sent for tissue nutrient analysis (described below).

### 2.3 Tissue nutrient analysis

Dried shoots were ground using a mortar and pestle to pass through a 1-mm sieve. Samples were sent to the Service Testing and Research Laboratory (STAR Laboratory, The Ohio State University, Wooster, OH, USA) for tissue nutrient analysis. Shoot tissue was digested with a microwave (Mars 6.0 Microwave Digestion System; CEM Corporation, Matthews, NC, USA). The total concentration of phosphorus (P), potassium (K), aluminum (Al), boron (B), calcium (Ca), copper (Cu), iron (Fe), magnesium (Mg), manganese (Mn), molybdenum (Mo), sodium (Na), sulfur (S), and zinc (Zn) was obtained using inductively coupled plasma optical emission spectroscopy (Agilent 5110 ICP-OES; Agilent Technologies, Santa Clara, CA, USA). For total nitrogen (TN) analysis, tissue samples were sent to Brookside Laboratories Inc. (New Bremen, OH, USA). The automated combustion method (B – 2.20) was conducted to quantify TN (Miller et al., 2013) with a C/N analyzer (Carlo Erba NA-1500; Carlo Erba Instruments, Milan, Italy).

### 2.4 Substrate, rhizosphere, and endosphere sampling for microbiome analysis

About 40 mL of bulk substrate was collected into a 50-mL centrifuge tube before seed sowing. At the end of the experiment, the unplanted pots were processed by discarding the top 1 cm and homogenizing the remaining substrate. Homogenized substrate was then collected into a 50-mL centrifuge tube.

To collect rhizosphere and endosphere samples, the root system was removed from the pot and gently shaken to remove all substrate loosely attached to the roots. The remaining substrate was set aside for later processing. Approximately 2.5 g root tissue (primarily secondary roots) was collected in 50-mL centrifuge tubes filled with 25 mL of phosphate buffered saline (per Liter: 8 g NaCl, 0.2 g KCl, 1.42 g Na_2_HPO_4_, 0.24 g KH_2_PO_4_, and 200 μL surfactant-Tween 20; pH 7.4). The rhizosphere substrate was washed off the roots by shaking them for 15 min at room temperature on an orbital shaker at 300 rpm (Richter-Heitmann et al., 2016). The washed roots were transferred to a new 50-mL centrifuge tube and set aside for later processing. The tube containing the rhizosphere wash was centrifuged (5,000 rpm for 20 min) to collect rhizosphere substrate in the bottom of the tube, and the supernatant was discarded (Wei et al., 2019). The resulting pellet was the rhizosphere sample and was stored at −80 °C until further processing (French et al., 2020). Twenty-five milliliters of 5% NaOCl was added to the washed roots in the 50-mL centrifuge tubes and incubated for 2 min. After incubation, roots were washed 3 times with autoclaved distilled deionized water. The surface sterilized roots (~300 mg) were stored in 2-mL microcentrifuge tubes at −80 °C until further processing (endosphere samples). Frozen samples (substrate and endosphere) were vacuum freeze-dried at −15 °C for 24 h in a freeze-dryer (Harvest Right, Salt Lake City, UT, USA). The freeze-dried samples were stored at −80 °C until DNA extraction. Seven rhizosphere substrate samples and seven endosphere samples were collected from each petunia cultivar (Unplanted, Picobella, and Wave) and each fertilizer treatment (low, optimum, and high).

### 2.5 Substrate nutrient analysis

Once all samples were collected for microbiome analysis, 200 mL of substrate was collected from the remaining substrate in each pot, placed in a zip top plastic bag, and stored at 5 °C until further processing. Samples were prepared using the 1:1 water extraction protocol adapted from Altland and Owen (2024). Briefly, 200 mL deionized water was added to the bagged substrate sample and incubated for 1 h at room temperature (Altland and Owen, 2024). After incubation, the bottom corner of the zip top bag was pierced to collect the liquid extract in a 50-mL centrifuge tube. The electrical conductivity (EC) and pH of the extract were measured with the LAQUA twin B-771 EC meter and the LAQUA twin pH11 meter (Horiba Ltd., Kyoto, Japan). The remaining extract was filtered (~35 mL) using a 0.45 μm nylon filter and analyzed for nutrient content. Total organic carbon (TOC) and total dissolved nitrogen (TN) were measured using a Shimadzu TOC-VCPH total organic carbon analyzer with TNM-1 TN measuring unit (Shimadzu Scientific Instruments, Columbia, MD, USA). Ten milliliters of the filtered extract was sent to the STAR Laboratory (The Ohio State University) for nutrient analysis. The total concentrations of P, K, Ca, Mg, S, Al, B, Cu, Fe, Mn, Mo, Na, and Zn were measured on an Agilent 5110 ICP-OES system (Agilent Technologies).

### 2.6 DNA extraction and sequencing

DNA was isolated from 125 mg of substrate (unplanted and rhizosphere) and ~20 mg of freeze-dried root (endosphere) samples using the DNeasy PowerSoil Pro Kit (Qiagen, Germantown, MD, USA). Samples were homogenized using a FAST-Prep25 (MP Biomedicals, Irvine, CA, USA). Substrate (bulk, unplanted, and rhizosphere) was placed into the PowerBead-Pro-Tube with the standard beads supplied by the manufacturer and homogenized twice at 5 m·s^−1^ for 30 s, with a 60 s incubation in ice between runs. Freeze-dried root samples were placed into PowerBead-Pro-Tubes, where the standard beads were replaced with two metallic beads and homogenized at 6 m·s^−1^ for 40 s. Then, 800 μL of Solution CD1 was added to each tube, followed by a 10 min incubation at 65 °C. After incubation, samples were homogenized again at 5 m·s^−1^ for 30 s. ZymoBIOMICS Microbial Community Standard (Zymo Research, Irvine, CA, USA) was included as a positive control. Seventy-five microliters of the community standard and 800 μL of solution CD1 were transferred into the PowerBead-Pro-Tube with standard beads and homogenized twice at 5 m·s^−1^ for 30 s, with a 60 s incubation in ice between runs. For the blank negative controls, 800 μL of solution CD1 was placed into the PowerBead-Pro-Tube with standard beads or metallic beads and homogenized twice at 5 m·s^−1^ for 30 s, with a 60 s incubation in ice between runs. All downstream steps were performed according to the manufacturer’s instructions.

DNA concentrations and quality were assessed using a NanoDrop ND-100 spectrophotometer (Thermo Fisher Scientific, Waltham, MA, USA). Extracted DNA was stored at −80 °C until sequencing. DNA concentrations were adjusted to 5 ng·μL^−1^, and 25 μL of the diluted samples was sent for 16S rRNA amplicon sequencing to the Molecular and Cellular Imaging Center (MCIC) (The Ohio State University, Wooster, OH, USA). The variable region 4 of the 16S rRNA gene was amplified using the 515F (5′-GTGCCAGCMGCCGCGGTAA-3′) and 806R (5′-GGACTACHVGGGTWTCTAAT-3′) primers (Caporaso et al., 2012). The resulting libraries were sequenced to obtain 300-bp paired-end reads using the Illumina NextSeq1000 platform (Illumina, San Diego, CA, USA). Raw demultiplexed reads are accessible in the short-read archive (SRA) under the BioProject PRJNA1282715.

### 2.7 Statistical analysis and bioinformatics

Dry weights, digital biomass, GLI, NDVI, NPCI, PSRI, and nutrient concentrations (substrate and tissue) were analyzed via one-way analysis of variance (ANOVA) according to the following model: Y = block + treatment. Normality and homoscedasticity were checked on the residuals. If the F-test was significant (*p* ≤ 0.05), multiple comparisons were conducted according to Tukey’s honestly significant difference (HSD) test (*p* ≤ 0.05) using the emmeans v1.10.7 package. All statistical analyses were performed using R v4.3.1 (R Core Team, 2022).

Raw reads were quality checked using FastQC (Andrews, 2010). Cutadapt v4.6 (Martin, 2011) was used to trim primer and adapter sequences and to remove reads containing poly-G and poly-A sequences longer than 20 and 40 bp, respectively. Further read processing of the sequences was conducted in R v4.3.1 according to the DADA2 (v1.30.0) pipeline for amplicon sequence variant (ASV) inference (Callahan et al., 2016). Taxonomic classification was performed using the SILVA (v138.2) database and the Naïve Bayes classifier. Potential contaminant sequences were removed using the microDecon v1.0.2 package (McKnight et al., 2019). Data cleaning, which included removing singletons, chloroplast and mitochondrial sequences, and rarefaction were conducted using the phyloseq v1.48.0 package. Finally, ASVs present in less than 5% of the samples were removed. A total of 23,970,695 high-quality read sequences and 3,012 ASVs were obtained.

Due to variation in sequencing depth across sample types, four separate datasets were created to enable appropriate analysis. The complete-normalized dataset was created using total sum scaling (TSS). The complete-rarefied dataset was generated by rarefying all samples to 1,000 reads. The substrate-rarefied dataset that included only substrate samples (bulk substrate, unplanted substrate, and rhizosphere) was rarefied to 206,927 reads, and the endosphere-rarefied dataset that included only endosphere samples was rarefied to 835 reads. The complete-normalized dataset was used to calculate the core microbiome across fertilizer rates for each petunia cultivar and to identify genera shared and unique across unplanted substrate, the rhizosphere, and endosphere. The complete-rarefied dataset was used for alpha and beta diversity analyses across all sampling compartments (bulk substrate, unplanted substrate, rhizosphere, and endosphere), and the substrate-rarefied and endosphere-rarefied datasets were used to assess the effects of fertilizer rate and cultivar.

The core bacteriome across fertilizer rate for each cultivar and sampling compartment was identified with the microbiome v1.26.0 package. Core taxa were defined as those with a relative abundance of at least 0.1% (detection threshold = 0.001) and present in 90% of the samples within each group (prevalence threshold = 0.90) (Neu et al., 2021). The core bacteriome of each cultivar and compartment combination were compared and visualized with a heatmap using the ggplot2 v3.5.2 package.

Alpha diversity, beta diversity, and differential abundance analysis were conducted using the rarefied datasets using the phyloseq v1.48.0 and microeco1.15.0 packages. Richness and Shannon index were calculated to evaluate alpha diversity. Differences between treatments were tested using the non-parametric Kruskal-Wallis test followed by Dunn’s Test for Multiple Comparisons (*α* = 0.05). Beta diversity was calculated using Bray & Curtis dissimilarities and visualized using a principal coordinate analysis (PCoA) plot. Differences in community composition between treatments were tested using the permutational analysis of variance (PERMANOVA) using the adonis function in the vegan v2.6–10 package (Oksanen et al., 2025). Post-hoc pair-wise comparisons were performed using the pairwiseAdonis v0.4.1 package (Martinez Arbizu, 2020) and *p*-values were adjusted for multiple comparisons using the Benjamini-Hochberg procedure (Benjamini and Hochberg, 1995). Substrate mineral nutrient analysis data was fitted onto the PCoA ordinations (1 and 2) for each sample type (unplanted, rhizosphere, or root) using the env_fit() function in the vegan package. Differential abundance analysis was conducted at phylum and genus levels to identify enriched or depleted genera across fertilization rates for each cultivar and sampling compartment. Differential abundance analysis was conducted using the Kruskal-Wallis test followed by Dunn’s test. The *p*-values were adjusted for multiple comparisons using the Benjamini-Hochberg procedure (Benjamini and Hochberg, 1995). LEfSe (Linear discriminant analysis Effect Size) was used to identify biomarkers (i.e., taxa that explain differences between experimental conditions) (Segata et al., 2011). Finally, differentially abundant phylum and genera identified by Kruskal-Wallis test were correlated to substrate mineral analysis. The results were visualized using a heatmap using the ggplot2 v3.5.2 package.

## 3 Results

### 3.1 Fertilizer rate affects petunia growth and flowering

**Figure 1:**
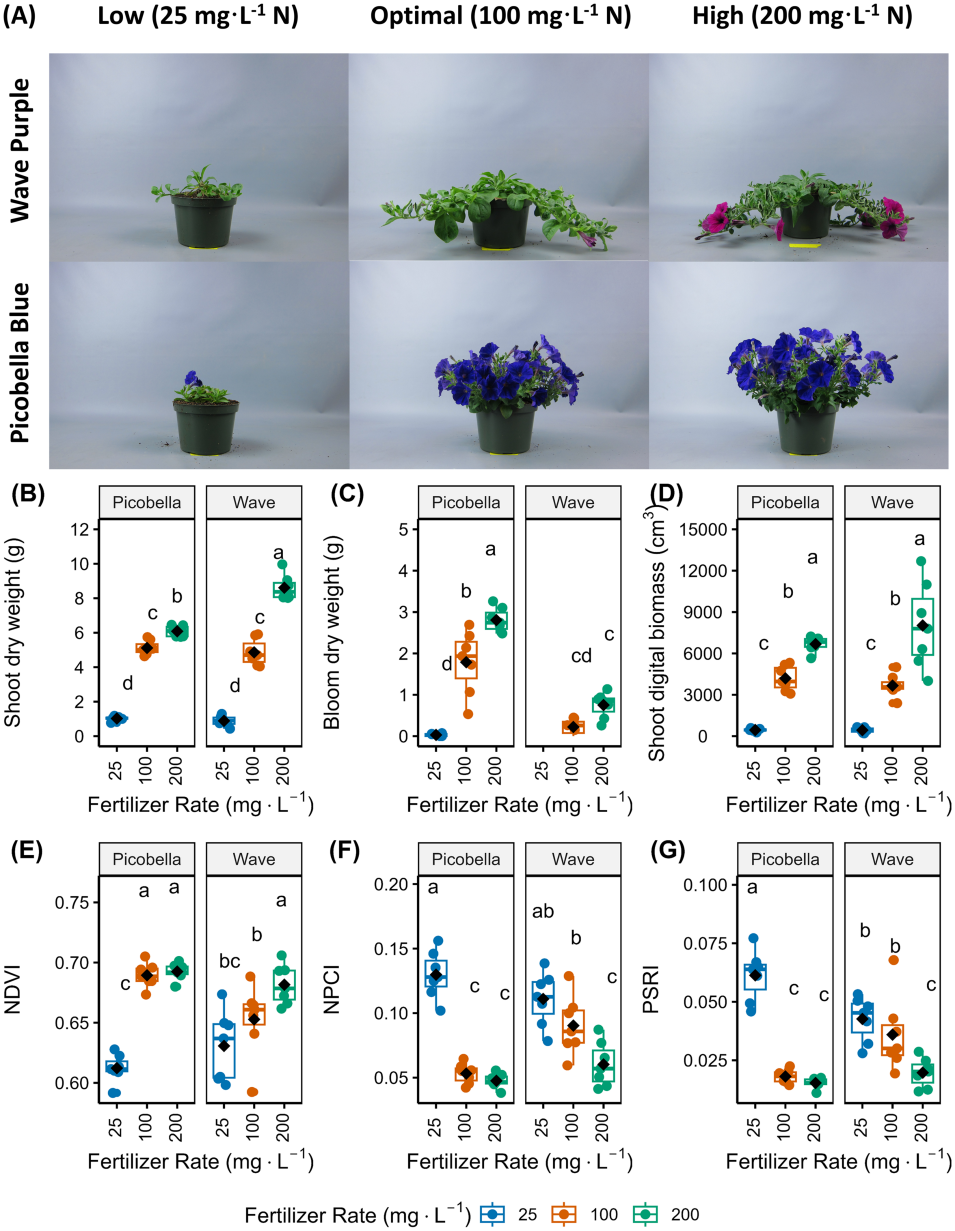
Effect of fertilizer rate on growth and physiological responses of two petunia cultivars. Representative pictures of petunia ‘Picobella Blue’ and ‘Wave Purple’ grown under three fertilizer rates (25, 100, and 200 mg·L^−1^ N) for 7 weeks **(A)**. Fertilizer solutions were prepared with a 20N–1.3P–15.8K Petunia FeED water soluble fertilizer. Boxplots showing the effects of fertilizer rate on shoot dry weight (only foliage) **(B)**, bloom (buds and flowers) dry weight **(C)**, shoot digital biomass (only foliage) **(D)**, normalized difference vegetation index (NDVI) **(E)**, normalized pigment chlorophyll index (NPCI) **(F)**, and plant senescence reflectance index (PSRI) **(G)**. Each boxplot displays the median (center line), interquartile range (box), mean (black diamonds), and individual data points (*n* = 7). Different lowercase letters indicate statistically significant differences based on Tukey’s honestly significant difference (HSD) test (*p* ≤ 0.05).

Picobella and Wave petunias fertilized with 200 mg·L^−1^ N were larger than petunias fertilized with either 100 mg·L^−1^ N or 25 mg·L^−1^ N. Petunias (Picobella and Wave) fertilized with 100 mg·L^−1^ N were larger than petunias fertilized with 25 mg·L^−1^ N (Figures 1A,B,D). The shoot dry weight (DW) and shoot digital biomass of petunias fertilized with 200 mg·L^−1^ N were higher than those of petunias fertilized with 100 or 25 mg·L^−1^ N, and shoot DW and shoot digital biomass of petunias fertilized with 100 mg·L^−1^ N were higher than those of petunias fertilized with 25 mg·L^−1^ N (Figures 1B,D). The dry weight of blooms (buds and flowers) from Picobella was the highest at 200 mg·L^−1^ N, followed by 100 mg·L^−1^ N, and then 25 mg·L^−1^ N. In contrast, Wave petunias fertilized with 25 mg·L^−1^ N did not flower, and there were no significant differences in bloom dry weight between the 100 and 200 mg·L^−1^ N fertilizer rates (Figure 1C).

Vegetation indices measured by the TraitFinder provide information about plant health. For GLI, we did not observe significant differences between fertilizer rates in any cultivar (Supplementary Figure S1). The NDVI of Picobella fertilized with 100 and 200 mg·L^−1^ N were not different, and both were higher than the NDVI measured from Picobella fertilized with 25 mg·L^−1^ N. In contrast, only the NDVI of Wave fertilized with 200 mg·L^−1^ N was higher than the NDVI of Wave fertilized with 25 mg·L^−1^ N (Figure 1E).

The NPCI of Picobella fertilized with 100 and 200 mg·L^−1^ N were not different, but both were lower than the NPCI of Picobella fertilized with 25 mg·L^−1^ N. In contrast, only the NPCI of Wave fertilized with 200 mg·L^−1^ N was lower than the NPCI of Wave fertilized with 25 mg·L^−1^ N (Figure 1F). A similar trend was observed for PSRI. Picobella fertilized with 100 or 200 mg·L^−1^ N had lower PSRI than Picobella fertilized with 25 mg·L^−1^ N, and only Wave fertilized with 200 mg·L^−1^ N had lower PSRI than Wave fertilized with 25 mg·L^−1^ N (Figure 1G).

In agreement with vegetation indices, Picobella showed the highest proportion of green canopy when they received 100 or 200 mg·L^−1^ N, and Wave had the highest proportion of green canopy only when fertilized with 200 mg·L^−1^ N. In addition, both petunia cultivars had the highest proportion of yellow canopy when fertilizer with 25 mg·L^−1^ N (Supplementary Figure S1).

### 3.2 Fertilizer rate influences tissue nutrient concentrations

Macronutrient concentrations within the petunia shoot tissue were influenced by fertilizer rates. Picobella fertilized with 200 mg·L^−1^ N had the highest N tissue concentration. In contrast, Wave fertilized with 100 or 200 mg·L^−1^ N had similar N tissue concentration levels, but higher than with 25 mg·L^−1^ (Table 1). Phosphorus tissue concentration in vegetative tissue increased as fertilizer rates increased. Petunias (Picobella or Wave) fertilized with 200 mg·L^−1^ N had the highest P tissue concentration (Table 1). In Picobella, K tissue concentration in 200 mg·L^−1^ N fertilized petunias was higher than in petunias fertilized with lower rates (25 and 100 mg·L^−1^ N). In contrast, there were no differences in K tissue concentrations between fertilizer rates in Wave (Table 1). Calcium tissue concentration decreased as the fertilizer rate increased, and the Ca tissue concentrations in Picobella were consistently higher than in Wave across all fertilizer rates. Within each cultivar, the 25 mg·L^−1^ N rate had the highest Ca concentration (Table 1). In Picobella, the Mg tissue concentration also decreased as the fertilizer rate increased. There were no differences in Mg concentrations between fertilizer rates in Wave (Table 1). Sulfur tissue concentration increased as the fertilizer rate increased only in Picobella (Table 1).

**Table 1:** Mineral macronutrient concentrations in dry shoot tissue of petunia.

Cultivar	Fertilizer rate (mg·L^−1^)	N (μg·g^−1^)	P (μg·g^−1^)	K (μg·g^−1^)	Ca (μg·g^−1^)	Mg (μg·g^−1^)	S (μg·g^−1^)
Picobella	25	38,571 ± 538 c	1,022 ± 35 d	27,961 ± 1,154 b	19,621 ± 544 a	15,584 ± 527 a	2,952 ± 146 d
Picobella	100	42,100 ± 1,337 c	2,118 ± 89 c	27,287 ± 868 b	17,380 ± 1,033 b	12,754 ± 695 b	3,897 ± 196 c
Picobella	200	52,500 ± 894 b	4,092 ± 128 a	34,544 ± 582 a	15,346 ± 362 b	10,303 ± 183 c	5,582 ± 207 a
Wave	25	42,686 ± 1,584 c	1,735 ± 213 c	35,043 ± 2,302 a	12,610 ± 285 c	9,830 ± 309 c	4,364 ± 243 bc
Wave	100	54,614 ± 1,869 ab	3,231 ± 258 b	36,009 ± 1,406 a	11,758 ± 582 cd	10,644 ± 634 c	4,927 ± 188 ab
Wave	200	59,157 ± 1,472 a	3,998 ± 92 a	38,947 ± 1,613 a	10,159 ± 212 d	9,471 ± 314 c	5,079 ± 76 ab
Recommended^i^		38,500–76,000	4,700–9,300	31,300–66,500	12,000–28,100	3,600–13,700	3,300–8,000
Recommended^ii^		44,200–59,900	4,500–7,800	44,900–66,300	10,900–18,900	5,200–9,700	3,300–6,100

i Recommended sufficiency ranges suggested by Gibson et al. (2007).

ii Recommended sufficiency ranges suggested by Veazie et al. (2025).

Petunia ‘Picobella Blue’ and ‘Wave Purple’ were grown under three fertilizer rates (25, 100, and 200 mg·L^−1^ N) for 7 weeks.

Means followed by the same letter are not significantly different according to Tukey’s HSD test (*p* ≤ 0.05).

Micronutrient concentration in the shoot tissue depended on cultivar and fertilizer rate. Differences in B tissue concentrations across fertilizer rates were only observed in Picobella. Boron concentration decreased as the fertilizer rate increased (Table 2). Similarly, differences in Cu tissue concentrations across fertilizer rates were only observed in Picobella, but Cu concentration increased as the fertilizer rate increased (Table 2). In contrast, differences in Fe tissue concentrations were only observed in Wave. Iron concentration decreased as the fertilizer rate increased. Manganese, Na, and Zn showed a similar pattern, tissue concentration levels were higher in petunias (Picobella and Wave) fertilized with 25 mg·L^−1^ N than those fertilized with higher rates (100 and 200 mg·L^−1^ N). Manganese, Na, and Zn tissue concentrations between the 100 and 200 mg·L^−1^ N rates were not different (Table 2).

**Table 2:** Mineral micronutrient concentrations in dry shoot tissue of petunia.

Cultivar	Fertilizer rate (mg·L^−1^)	B (μg·g^−1^)	Cu (μg·g^−1^)	Fe (μg·g^−1^)	Mn (μg·g^−1^)	Na (μg·g^−1^)	Zn (μg·g^−1^)
Picobella	25	57.9 ± 2.3 a	1.2 ± 0.1 b	629.3 ± 41.8 ab	486.7 ± 19.5 a	13,451.4 ± 403.7 a	106.2 ± 4.7 b
Picobella	100	44.4 ± 1.6 b	1.3 ± 0.1 b	798.9 ± 74.1 a	331.1 ± 24.3 b	11,652.9 ± 391.9 ab	77.6 ± 3.6 c
Picobella	200	36.1 ± 0.6 c	1.7 ± 0.1 a	841.2 ± 47.6 a	345.1 ± 10.6 b	8,513.9 ± 209.3 cd	73.1 ± 2.2 cd
Wave	25	38.1 ± 3.1 bc	1.2 ± 0.1 b	829.7 ± 76.6 a	359.8 ± 31.9 b	9,619.9 ± 729.3 bc	143 ± 7.5 a
Wave	100	39 ± 1.5 bc	1.2 ± 0.1 b	654.2 ± 40.2 ab	203.5 ± 8.7 c	7,113.9 ± 773.7 d	63.8 ± 2.2 cd
Wave	200	34.6 ± 1 c	1.3 ± 0.1 b	451.7 ± 50.5 b	144.7 ± 9.5 c	6,870.1 ± 667.6 d	59 ± 2.2 d
Recommended^i^		18–43	3–19	84–168	44–177		44–177
Recommended^ii^		19.4–33.8	3.8–10.6	76.1–123.0	44.2–108.4		38.5–73.3

i Recommended sufficiency ranges suggested by Gibson et al. (2007).

ii Recommended sufficiency ranges suggested by Veazie et al. (2025).

Petunia ‘Picobella Blue’ and ‘Wave Purple’ were grown under three fertilizer rates (25, 100, and 200 mg·L^−1^ N) for 7 weeks.

Means followed by the same letter are not significantly different according to Tukey’s HSD test (*p* ≤ 0.05).

### 3.3 Fertilizer rate modulates substrate chemical properties

Substrate chemical properties were significantly influenced by both fertilizer rate and plant presence (Supplementary Figure S2). The substrate pH decreased in response to increasing fertilization rate only when plants were present (Supplementary Figure S2A). The lowest pH in Picobella (4.16) and Wave (4.15) was observed at 200 mg·L^−1^ N. However, unplanted pots fertilized with the same N rate had a higher pH of 4.51. In contrast, EC increased consistently with fertilizer rate in both planted and unplanted substrate (Supplementary Figure S2B). Compared to unplanted substrate, the TOC of the substrate was higher in planted pots. In addition, fertilization rate did not affect TOC (Supplementary Figure S2C).

Total N, P, and K consistently increased with fertilizer rate only in the unplanted substrate (Supplementary Figures S2D–F). For the substrate from Picobella and Wave petunias, the N and K concentrations at 25 and 100 mg·L^−1^ N were the same, while the 200 mg·L^−1^ N rate was the highest. Phosphorus concentrations were not different across fertilizer rates in planted pots. Both Picobella and Wave showed consistently low TN, P, and K in the substrate across fertilizer rates, suggesting active plant uptake.

The concentrations of Ca, Mg, and S were not influenced by fertilizer rate in unplanted substrate. However, they consistently increased with fertilizer rate in planted substrate (for both petunia cultivars) (Supplementary Figures S3A–C). In unplanted and planted substrate, clear differences in B concentrations were observed only between 25 and 200 mg·L^−1^ (Supplementary Figure S3D). Similarly, significant differences in Fe and Mn concentrations were observed between 25 and 200 mg·L^−1^ in unplanted substrate (Supplementary Figures S3E,F). However, for the planted substrate, clear separations of Fe and Mn concentrations between each fertilizer rate were observed.

Sodium concentrations were not different across fertilizer rates in unplanted substrate. However, a distinct separation between fertilizer rates was observed in planted substrate, where Na concentrations increased with higher fertilizer rates (Supplementary Figure S3G). There were no differences in Zn concentrations across treatments (Supplementary Figure S3H).

### 3.4 The bacteriome undergoes significant changes across the substrate–rhizosphere–endosphere continuum

**Figure 2:**
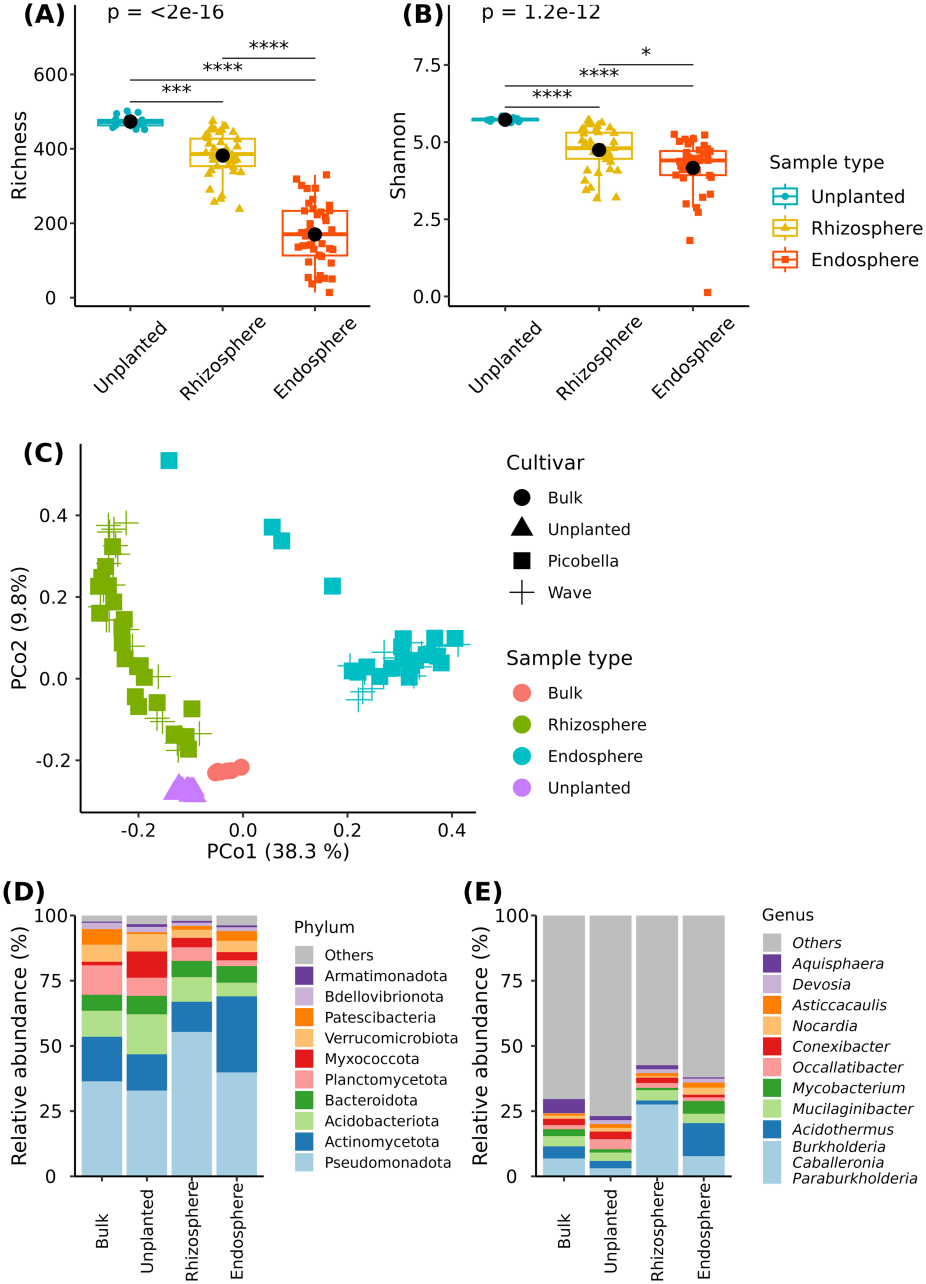
Bacteriome of bulk substrate, unplanted substrate, rhizosphere, and endosphere samples. Rhizosphere and endosphere were collected from petunia ‘Picobella Blue’ and ‘Wave Purple’ grown under three fertilizer rates (25, 100, and 200 mg·L^−1^ N) for 7 weeks. Unplanted substrate also received three fertilization rates for 7 weeks. Boxplots showing richness **(A)** and Shannon index **(B)** across sampling compartments. Each boxplot displays the median (center line), interquartile range (box), mean (black diamonds), and individual data points (*n* = 7). Asterisks indicate statistically significant differences based on Dunn’s tests (*p ≤ 0.05, **p ≤ 0.01, ***p ≤ 0.001, ****p ≤ 0.0001). Principal coordinate analysis based on the Bray–Curtis distance **(C)**. Relative abundance of major bacterial phyla **(D)** and genera **(E)** across sampling compartments.

We first examined differences across sampling compartments (unplanted substrate, rhizosphere, and endosphere). Unplanted substrate was more diverse than the rhizosphere or the endosphere. The unplanted substrate had more ASVs and higher Shannon index values than rhizosphere and endosphere samples. In addition, the rhizosphere samples had more ASVs and had higher Shannon index than the endosphere samples (Figures 2A,B).

Differences in community composition across sample types were visualized using principal coordinate analysis on a Bray–Curtis dissimilarity matrix. We observed clustering of samples according to the sampling compartment (bulk substrate, unplanted substrate, rhizosphere, and endosphere) (Figure 2C). The first two principal coordinates accounted for 48.1% of the total variation in the dataset. Bulk, unplanted, and rhizosphere samples separated from endosphere samples along the first principal coordinate (PCo1). Bulk, unplanted, and rhizosphere samples formed three distinct clusters separated from each other along the second principal coordinate (PCo2). Multivariate analysis of variance (PERMANOVA) showed a significant effect of sample type (*R*^2^ = 0.44, *p* = 0.001) on the bacterial community structure.

Our results indicated that the substrate serves as the main source of microbial diversity. We found 393 and 321 genera in the rhizosphere and endosphere samples, among those 385 (97.9%) and 312 (97.2%) were also found in unplanted samples (Supplementary Figure S4A). Genera *Luteibacter, Roseateles*, and *Microcella* were only found in the rhizosphere samples, while *Cutibacterium, Allomeiothermus, Xanthobacter, Methylerubrum*, and *Klebsiella* were only found in endosphere samples (Supplementary Figure S4B).

Across the sampling compartments (bulk substrate, unplanted substrate, rhizosphere, and endosphere), the relative abundance of 18 phyla was significantly different (Supplementary material 2). Among the differentially abundant phyla, Pseudomonadota and Actinomycetota accounted for about 50% (bulk substrate and unplanted) or more (rhizosphere and endosphere) of the community across compartments. The relative abundance of Pseudomonadota was the highest in the rhizosphere (55.34%), and there were no differences between bulk, unplanted, and endosphere compartments. Interestingly, the relative abundance of Actinomycetota was the highest in the endosphere (29.11%), the lowest in the rhizosphere (11.6%), and there were no differences between bulk and unplanted substrates (Figure 2D and Supplementary material 2). At the genus level, we found 106 differentially abundant genera across compartments (Supplementary material 2). Notably, the rhizosphere samples were dominated by genus *Burkholderia-Caballeronia-Paraburkholderia*, that reached a relative abundance of 27.57%. In contrast, endosphere samples were dominated by genus *Acidothermus* (12.64%) (Figure 2E and Supplementary material 2).

Finally, we also identified a potential core bacteriome. An ASV was considered core if it was found in 90% of samples and had a relative abundance >0.1%. In the rhizosphere, the identified core bacteriome grouped into 29 and 27 core genera in the rhizosphere of Picobella and Wave, respectively. In contrast, the identified core ASVs in the endosphere grouped into three and two genera in Picobella and Wave, respectively. *Nocardia* and *Legionella* were the only genera represented in the rhizosphere and endosphere of both petunia cultivars (Supplementary Figure S4C).

### 3.5 Fertilization modulates substrate bacteriome

**Figure 3:**
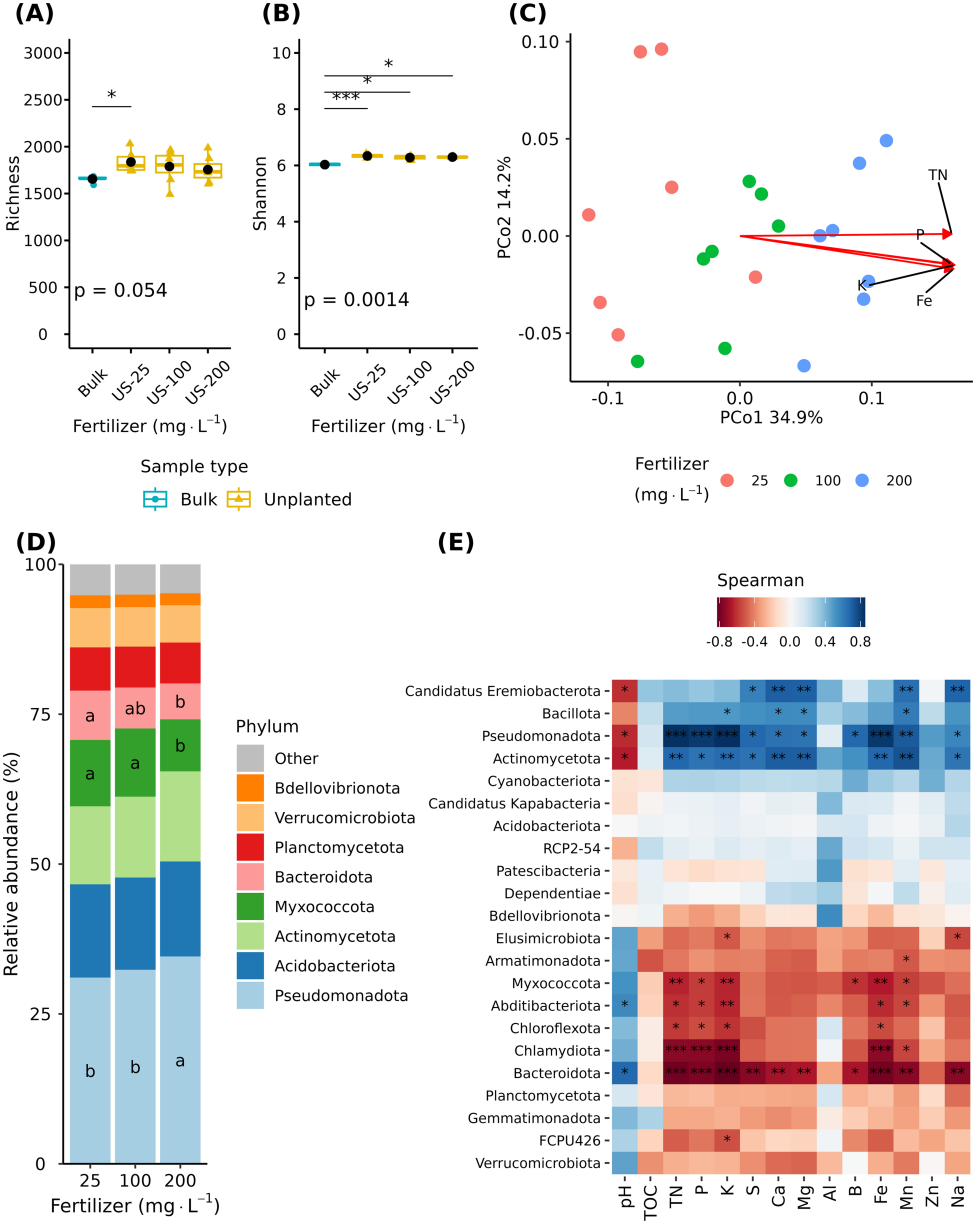
Effect of fertilizer rate on unplanted substrate bacteriome. Unplanted substrate received three fertilization rates (25, 100, and 200 mg·L^−1^ N) for 7 weeks. Boxplots showing richness **(A)** and Shannon index **(B)** across unfertilized bulk substrate (Bulk) and unplanted substrate (US). Each boxplot displays the median (center line), interquartile range (box), mean (black diamonds), and individual data points (*n* = 7). Asterisks indicate statistically significant differences based on Dunn’s tests (*p ≤ 0.05, **p ≤ 0.01, ***p ≤ 0.001, ***p ≤ 0.0001). Principal coordinate analysis based on the Bray–Curtis distance **(C)**. Arrows indicate significant correlations between substrate chemical properties and microbial community composition; only variables with an *r*^2^ > 0.75 are shown. Relative abundance of major bacterial phyla across fertilizer rates **(D)**. Spearman correlation between phylum abundance and substrate chemical properties **(E)**. Asterisks indicate statistically significant correlations (*p ≤ 0.05, **p ≤ 0.01, ***p ≤ 0.001).

Unplanted pots were included in the experimental design to test the effect of fertilizer alone on the substrate bacteriome without the influence of a plant. Although unplanted samples showed higher alpha diversity than bulk substrate, there was no difference between fertilizer rates (Figures 3A,B). In contrast, principal coordinate analysis (PCoA) revealed fertilizer rate driven clustering of samples (Figure 3C). Samples from the 25 and 200 mg·L^−1^ N are separated along the first principal coordinate (PCo 1), and samples from 100 mg·L^−1^ N rate were between the 25 and 200 mg·L^−1^ N (Figure 3C). In unplanted substrate, most of the mineral nutrients were significantly correlated with the bacterial community composition. *R*^2^ values ranged from 0.04 to 0.82. Total nitrogen, P, K, and Fe were the only nutrients with an *R*^2^ > 0.75 (Figure 3C). Total organic carbon, Al, and Zn were not correlated with bacterial community composition in unplanted substrate (Supplementary material 1). Multivariate analysis of variance (PERMANOVA) supported a significant effect of fertilizer (*R*^2^ = 0.34, *p* < 0.001) in the substrate bacteriome.

The substrate bacteriome contained 22 unique phyla. The eight more abundant phyla were Pseudomonadota, Acidobacteria, Actinomycetota, Myxococcota, Bacteroidota, Planctomycetota, Verrucomicrobiota, and Bdellovibrionota (Figure 3D). Among the more abundant phyla, Myxococcota and Bacteroidota decreased as the fertilizer rate increased. Moreover, they were negatively correlated with substrate nutrients (Figure 3E). In contrast, the relative abundance of Pseudomonadota and Actinomycetota increased as the fertilizer rate increased, and it was also positively correlated with substrate nutrients (Figure 3E; Supplementary material 3).

**Figure 4:**
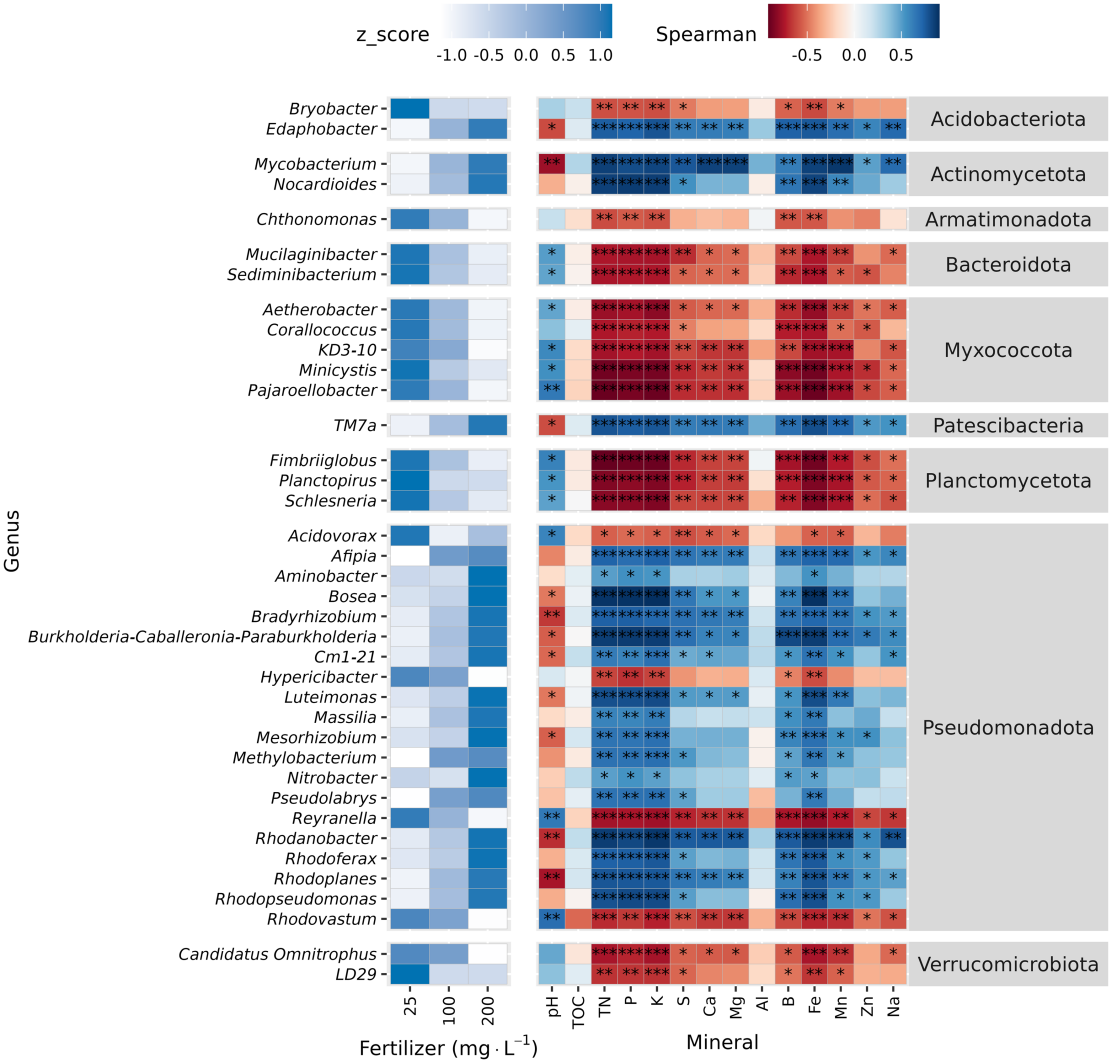
Differential abundance analysis and correlation analysis of unplanted substrate at genus level. Unplanted substrate received three fertilization rates (25, 100, and 200 mg·L^−1^ N) for 7 weeks. Relative abundance was normalized (Z-normalization) across fertilizer rates for each genus to facilitate visualization of increasing or decreasing trends. Heatmap displaying the normalized relative abundance of differentially abundant genera (left) across fertilizer rates and Spearman correlation between differentially abundant genera and substrate chemical properties (right). Asterisks indicate statistically significant correlations (*p ≤ 0.05, **p ≤ 0.01, ***p ≤ 0.001).

At genus level, Krustal-Wallis test identified 38 differentially abundant genera across fertilizer rates. The relative abundance of 20 genera increased with higher fertilizer rate, and 16 of these belong to phylum Pseudomonadota. In contrast, *Acidovorax*, *Hypericibacter*, *Reyranella*, and *Rhodovastum* also from Pseudomonadota were exceptions that decreased as the fertilizer rate increased. The relative abundance of *TM7a* (phylum Patescibacteria), *Edaphobacter* (Acidobacteriota), *Mycobacterium*, and *Nocardioides* (phylum Actinomycetota) also increased along with fertilizer rate (Figure 4 and Supplementary material 3). The other 18 genera decreased as the fertilizer rate increased and were negatively correlated with substrate nutrients (Figure 4). Total organic carbon and Al were not correlated with the relative abundance of differentially abundant taxa (Figure 4).

The LEfSe analysis identified seven phyla and 35 genera as potential biomarkers. Pseudomonatota and Actinomycetota were enriched at 200 mg·L^−1^ N. Bacteroidota, Myxococcota, Chlamydiota, Abditibacteriota, and FCPU426 were enriched at 25 mg·L^−1^ N (Supplementary Figure S5A). At genus level, *Burkholderia-Caballeronia-Paraburkholderia* and *Occallatibacter* were the top biomarkers for 200 mg·L^−1^ N, and *Mucilaginibacter* and *Bryobacter* were the top biomarkers for 25 mg·L^−1^ N (Supplementary Figure S5B).

### 3.6 Fertilization is the dominant factor shaping rhizosphere bacteriome in petunia

**Figure 5:**
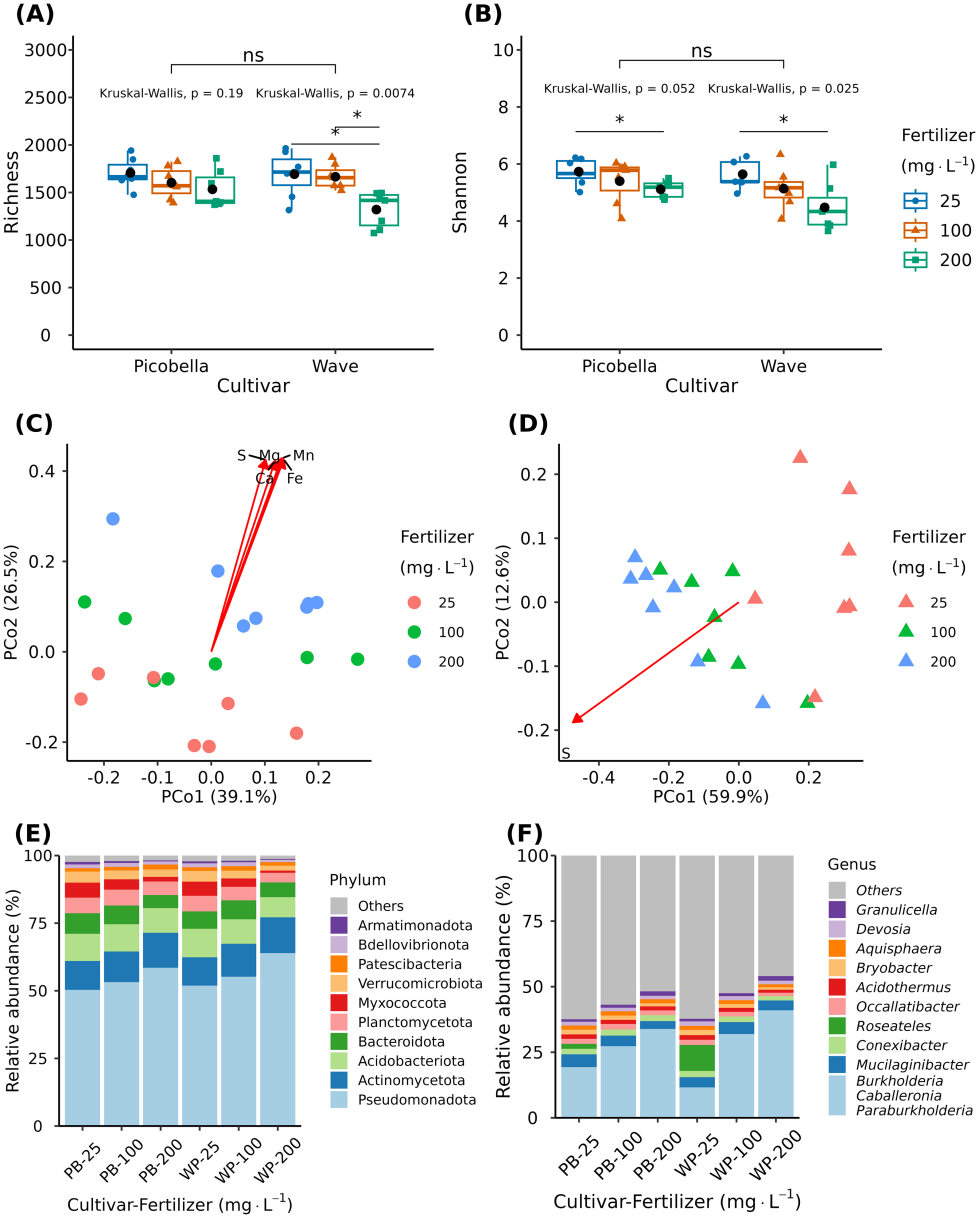
Effect of fertilizer rate on the rhizosphere bacteriome of petunia. Rhizosphere samples were collected from petunia ‘Picobella Blue’ and ‘Wave Purple’ grown under three fertilizer rates (25, 100, and 200 mg·L^−1^ N) for 7 weeks. Boxplots showing richness **(A)** and Shannon index **(B)** across fertilizer rates. Each boxplot displays the median (center line), interquartile range (box), mean (black diamonds), and individual data points (*n* = 7). Top brackets indicate significant differences between the two cultivars (ns *p* > 0.05). Kruskal-Wallis’ test indicates significant differences between fertilizer rates within each cultivar. Asterisks indicate statistically significant differences between fertilizer rates based on Dunn’s tests (*p ≤ 0.05, **p ≤ 0.01, ***p ≤ 0.001, ***p ≤ 0.0001). Principal coordinate analysis based on the Bray–Curtis distance in petunia ‘Picobella Blue’ **(C)** and ‘Wave Purple’ **(D)**. Arrows indicate significant correlations between substrate chemical properties and rhizosphere microbial community composition; only variables with an *r*^2^ > 0.75 are shown. Relative abundance of major bacterial phyla **(E)** and genera **(F)** across fertilizer rates.

Alpha diversity metrics of rhizosphere samples were not different among petunia cultivars (Figures 5A,B). However, we observed a fertilization effect on both cultivars. Richness and Shannon diversity decreased as the fertilizer rate increased (Figures 5A,B). Wave rhizosphere samples had lower richness at 200 mg·L^−1^ N than at 25 and 100 mg·L^−1^ N (Figure 5A). Both cultivars had lower Shannon diversity at 200 mg·L^−1^ N than at 25 mg·L^−1^ N (Figure 5B).

Multivariate analysis of variance (PERMANOVA) revealed a significant effect of fertilizer (*R*^2^ = 0.31, *p* < 0.0001) and cultivar (*R*^2^ = 0.05, *p* = 0.019), but their interaction effect was not significant (*R*^2^ = 0.06, *p* = 0.06). The fertilizer factor explained a higher proportion of the total variation in the data. Principal coordinate analysis revealed fertilizer driven clustering of samples for both cultivars (Figures 5C,D). The first two principal coordinates accounted for 65.6 and 72.5% of the variation in community composition of Picobella and Wave, respectively. Like the unplanted samples, most of the mineral nutrients were significantly correlated with the bacterial community composition. *R*^2^ values ranged from 0.04 to 0.80 and 0.25 to 0.77 in Picobella and Wave, respectively. In Picobella, Mg, Ca, S, Fe, and Mn had an *R*^2^ > 0.75 (Figure 5C). In Wave, only S had an *R*^2^ > 0.75 (Figure 5D). Total organic carbon, Al, and Zn were not correlated with Picobella bacterial composition (Supplementary material 1). Aluminum and Zn were not correlated with Wave bacterial community composition (Supplementary material 1).

The rhizosphere bacteriome contained 22 unique phyla. The 10 most abundant phyla were Pseudomonadota, Actinomycetota, Acidobacteria, Bacteroidota, Planctomycetota, Myxococcota, Verrucomicrobiota, Patescibacteria, Bdellovibrionota, and Armatimonadota (Figure 5E). The relative abundance of phyla Verrucomicrobiota, Armatimonadota, Chloroflexota, Elusimicrobiota, Myxococcota, and Gemmatimonadota were affected by fertilization treatment in both petunia cultivars. Acidobacteriota, Bdellovibrionota, and FCPU426 were affected only in Wave, and Bacteroidota was affected only in Picobella. The relative abundance of all significant phyla was reduced as the fertilizer rate increased (Figure 5E; Supplementary material 4).

**Figure 6:**
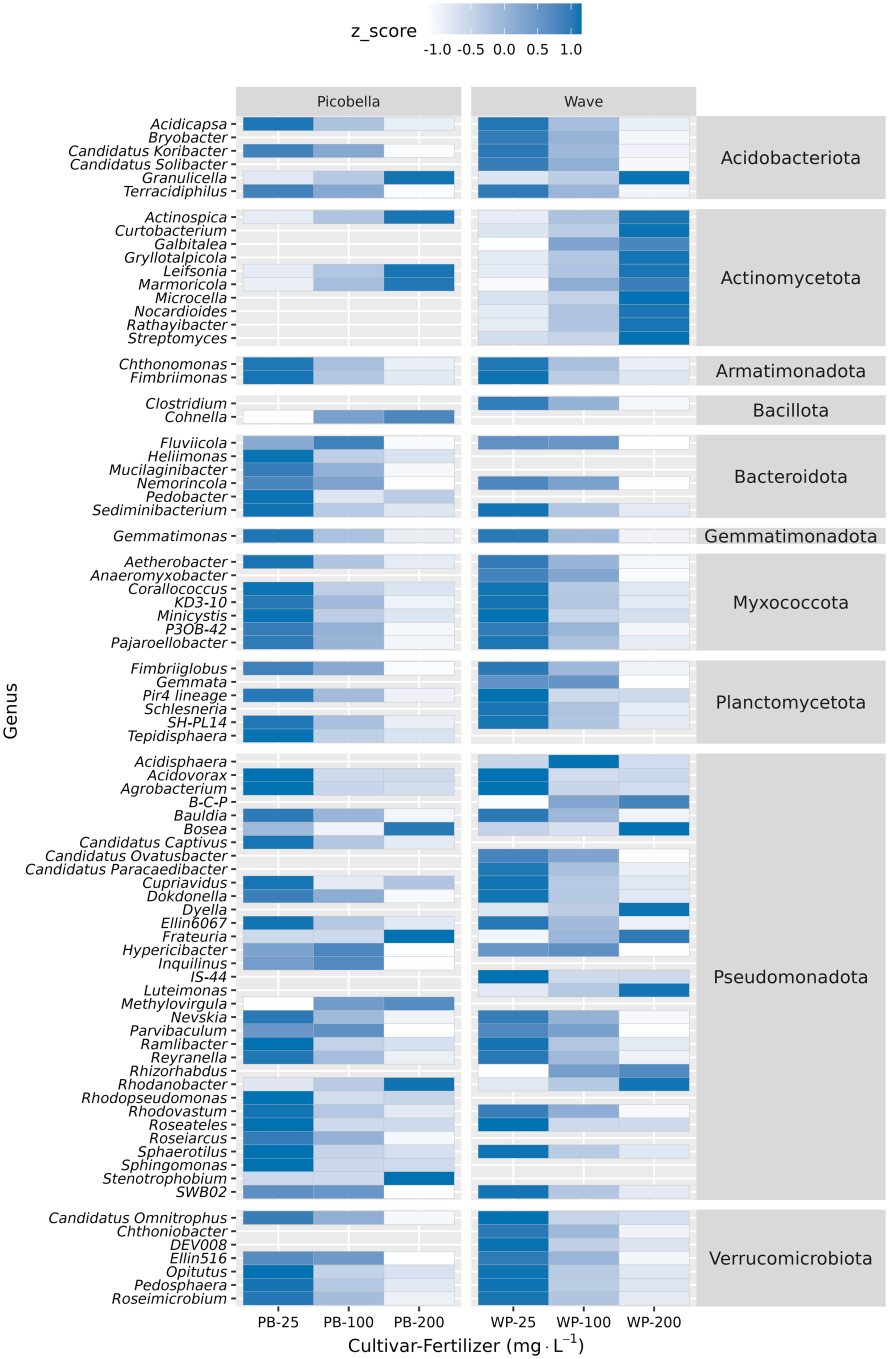
Differential abundance analysis of rhizosphere samples at genus level. Rhizosphere samples were collected from petunia ‘Picobella Blue’ and ‘Wave Purple’ grown under three fertilizer rates (25, 100, and 200 mg·L^−1^ N) for 7 weeks. Relative abundance was normalized (Z-normalization) across fertilizer rates for each genus to facilitate visualization of increasing or decreasing trends. Heatmap displaying the normalized relative abundance of differentially abundant genera across fertilizer rates. Burkholderia-Caballeronia-Paraburkholderia (B-C-P).

The 10 most abundant genera were *Burkholderia-Caballeronia-Paraburkholderia, Mucilaginibacter, Conexibacter, Roseateles, Occallatibacter, Acidothermus*, *Bryobacter*, *Aquisphaera, Devosia*, and *Granulicella* (Figure 5F). Krustal-Wallis test identified 57 genera in Picobella and 68 in Wave. Among the differentially abundant genera, 45 were identified in both cultivars. Differentially abundant genera from phylum Verrucomicrobiota (7), Planctomycetota (6), Myxococcota (7), Gemmatimonadota (1), Bacteriodota (6), and Armatimonadota (2) decreased in response to increasing fertilizer rate. In contrast, the 10 differentially abundant genera in phylum Actinomycetota increased with increasing fertilizer rates. Six genera from phylum Acidobacteria were influenced by fertilizer. *Acidicapsa, Bryobacter, Candidatus Koribacter, Candidatus Solibacter*, and *Terracidiphilus* decreased as fertilizer rate increased. Only *Granulicella* increased in response to increased fertilizer rate. Finally, within phylum Pseudomonadota, nine genera increased (*Burkholderia-Caballeronia-Paraburkholderia*, *Bosea*, *Dyella, Frateuria, Luteimonas, Methylovirgula, Rhizorhabdus, Rhodanobacter* and *Stenotrophobium*) and 24 genera decreased in response to increasing fertilizer rate (Figure 6; Supplementary material 4).

**Figure 7:**
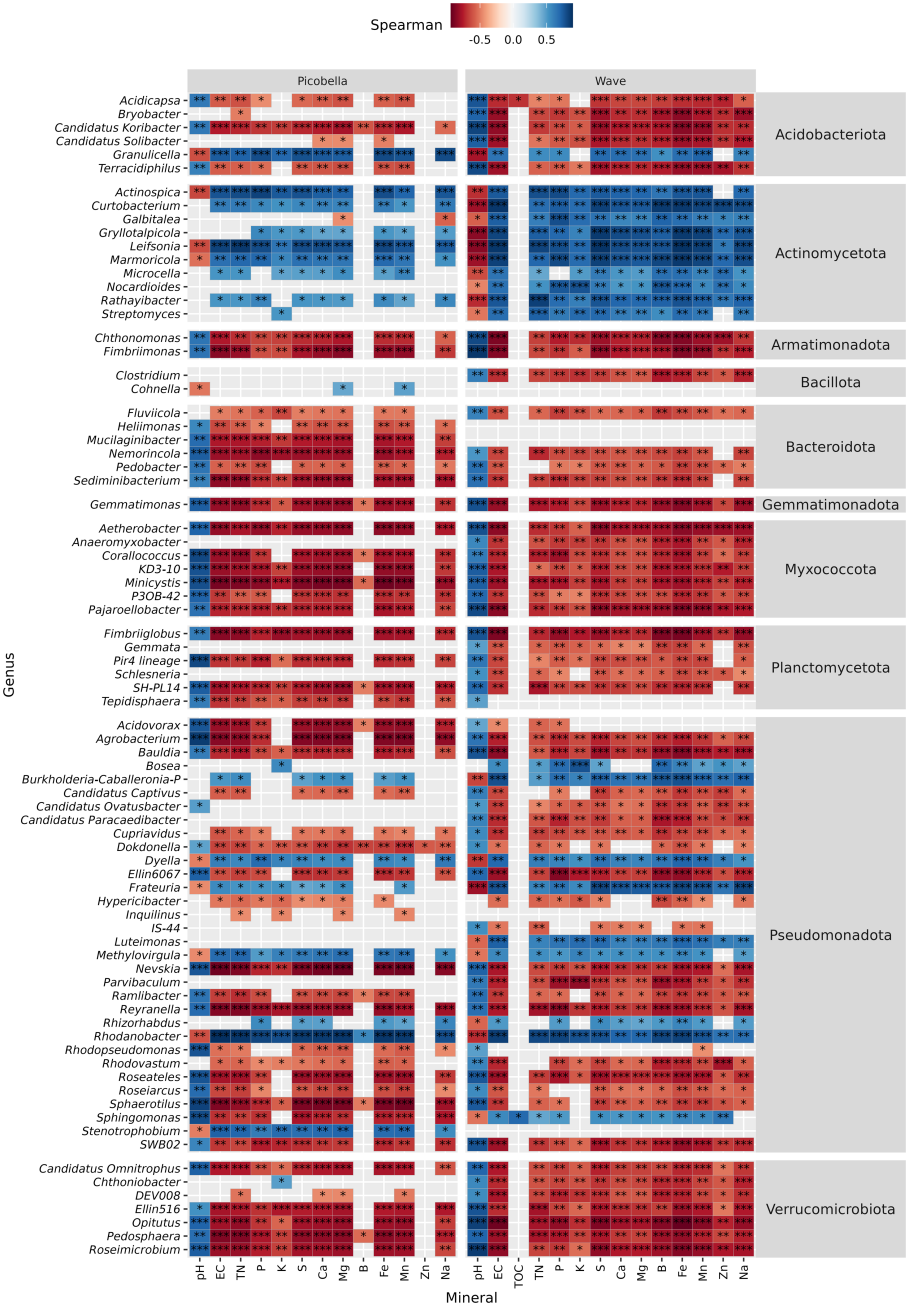
Petunia rhizosphere bacteriome. Spearman correlation between differentially abundant genera and substrate chemical properties. Asterisks indicate statistically significant correlations (*p ≤ 0.05, **p ≤ 0.01, ***p ≤ 0.001). Rhizosphere samples were collected from petunia ‘Picobella Blue’ and ‘Wave Purple’ grown under three fertilizer rates (25, 100, and 200 mg·L^−1^ N) for 7 weeks.

Significant genera that decreased in response to fertilization showed negative correlation with nutrient concentration but positive correlation with pH (Figure 7). In contrast, genera that increased in response to fertilization rate showed positive correlation with nutrient concentration but negative with pH. In Picobella, only a few genera showed significant association to B and Zn. Among differently abundant genera identified in Wave, only two correlated with TOC.

The LEfSe analysis identified nine phyla and 66 genera in Picobella, and 16 phyla and 56 genera in Wave as potential biomarkers (Supplementary Figures S6, S7). Pseudomonadota was enriched at 200 mg·L^−1^ N in both cultivars. At 25 mg·L^−1^ N, Myxococcota and Bacteroidota were the two top biomarkers in Picobella, and Myxococcota and Acidobacteriota were the top biomarkers in Wave (Supplementary Figure S6). At genus level, *Burkholderia-Caballeronia-Paraburkholderia* and *Dyella* were the two top biomarkers for 200 mg·L^−1^ N in Picobella, and *Burkholderia-Caballeronia-Paraburkholderia* and *Rhodanobacter* were the top biomarkers in Wave. At 25 mg·L^−1^ N, *Roseateles* and *Sphaerotilus* were the two top biomarkers in Picobella, while *Roseateles* and *Mucilaginibacter* were the top biomarkers in Wave (Supplementary Figure S7).

### 3.7 Cultivar and fertilizer rate modulate the petunia endosphere bacteriome

**Figure 8:**
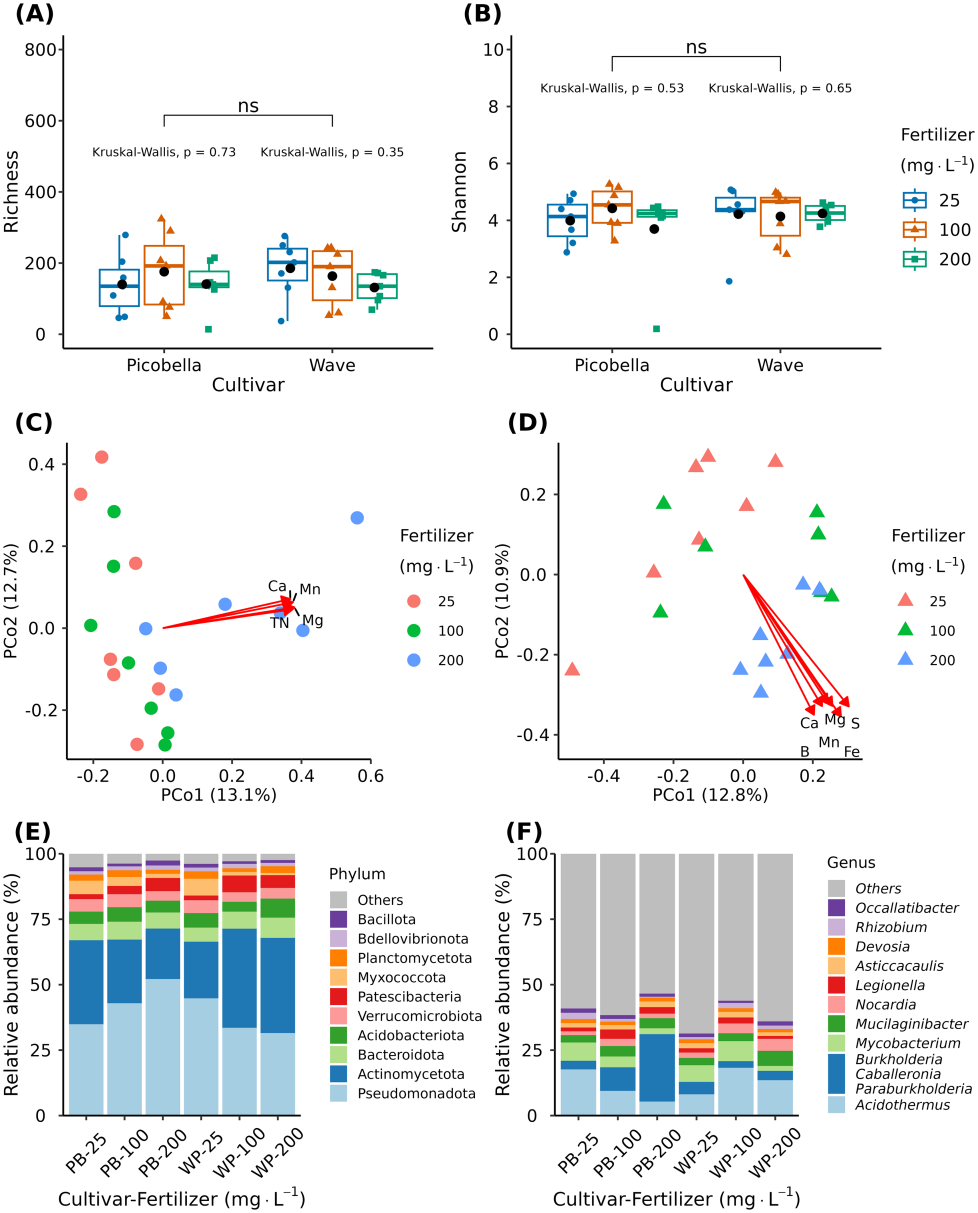
Petunia root endosphere bacteriome. Root endosphere samples were collected from petunia ‘Picobella Blue’ and ‘Wave Purple’ grown under three fertilizer rates (25, 100, and 200 mg·L^−1^ N) for 7 weeks. Boxplots showing richness **(A)** and Shannon index **(B)** across fertilizer rates. Each boxplot displays the median (center line), interquartile range (box), mean (black diamonds), and individual data points (*n* = 7). Top brackets indicate significant differences between the two cultivars (ns *p* > 0.05). Kruskal-Wallis test indicates significant differences between fertilizer rates within each cultivar. Principal coordinate analysis based on the Bray–Curtis distance in petunia ‘Picobella Blue’ **(C)** and ‘Wave Purple’ **(D)**. Arrows indicate significant correlations between substrate chemical properties and rhizosphere microbial community composition; only variables with an *r*^2^ > 0.55 are shown. Relative abundance of major bacterial phyla **(E)** and genera **(F)** across fertilizer rates.

In contrast to rhizosphere and substrate samples, the alpha diversity of endosphere samples was not different among petunia cultivars (Figures 8A,B). However, multivariate analysis of variance (PERMANOVA) revealed a significant effect of fertilizer (*R*^2^ = 0.085, *p* = 0.014) and cultivar (*R*^2^ = 0.031, *p* = 0.042) and their interaction (*R*^2^ = 0.061, *p* < 0.0001). However, our experimental variables explained only a small proportion of the total variation in the data. Principal coordinate analysis revealed fertilizer driven clustering of samples for both cultivars (Figures 8C,D). The first two principal coordinates accounted for 25.8 and 23.7% of the variation in community composition of Picobella and Wave, respectively.

Similar to unplanted and rhizosphere samples, most of the mineral nutrients were significantly correlated with the endosphere bacterial community composition. *R*^2^ values ranged from 0.03 to 0.59 and 0.11–0.72 in Picobella and Wave, respectively. In Picobella, TN, Mg, Ca, Fe, and Mn had an *R*^2^ > 0.5 (Figure 8C). Total organic carbon, P, Al, B, and Zn were not correlated with Picobella bacterial community composition (Supplementary material 1). In Wave, TN, Na, Mg, Ca, S, B, Fe, and Mn had an *R*^2^ > 0.5. Total organic carbon and Al were not correlated with Wave bacterial community composition (Supplementary material 1).

The endosphere bacteriome contained 22 unique phyla. The 10 most abundant phyla were Pseudomonadota, Actinomycetota, Bacteroidota, Acidobacteria, Verrucomicrobiota, Patescibacteria, Myxococcota, Planctomycetota, Bdellovibrionota, and Bacillota (Figure 8E). We did not find differentially abundant phyla between fertilization treatments in both petunia cultivars. The 10 most abundant genera were *Acidothermus*, *Burkholderia-Caballeronia-Paraburkholderia, Mycobacterium, Mucilaginibacter, Nocardia, Legionella, Asticcacaulis, Devosia, Rhizobium*, and *Occallatibacter* (Figure 8F). Similarly, we did not find differentially abundant genera across fertilizer rates. LEfSe analysis did not identify potential biomarkers.

## 4 Discussion

### 4.1 Plant–fertilizer interactions influence nutrient dynamics and pH in the rhizosphere

Among ornamental crops, petunias are known to respond well to higher fertilizer rates, and they are susceptible to Fe deficiency. To avoid Fe symptomatology the recommended substrate pH for both regular and wave petunias is 5.5–6.2. The recommended fertilizer rate for regular petunias is 150–200 mg·L^−1^ and for wave petunias is 200–250 mg·L^−1^ N (Gibson et al., 2007; Nau et al., 2021). In our evaluation, the best growth was observed at the highest fertilization rate (200 mg·L^−1^ N) for both petunia cultivars. However, Picobella (a regular petunia cultivar) fertilized with 100 and 200 mg·L^−1^ N showed similar performance across all health parameters evaluated. In contrast, Wave performed best when fertilized with 200 mg·L^−1^ N. These results highlight the distinct fertilizer requirements between cultivars, with Picobella benefiting from both moderate and high fertilization, while Wave required a high rate to achieve growth and health comparable to Picobella.

At 100 and 200 mg·L^−1^ N, the concentrations of N, P, and K in planted substrate were lower than in the unplanted substrate. These findings suggest active N, P, and K uptake by the petunia roots, while those nutrients accumulate in the absence of plants. In contrast, the concentrations of micronutrients (except B) in the rhizosphere were higher than in the unplanted substrate. Nutrients such as S, Ca, Mg, Fe, Mn, and Na are more soluble under acidic conditions, and their higher availability in the rhizosphere can be attributed to the lower pH. The fertilizer used in this project has a high proportion (40%) of ammonium meant to promote substrate acidification. The acidifying effect of ammonium fertilization is primarily driven by the release of H^+^ ions during microbial nitrification and ammonium uptake by roots (Argo and Fisher, 2003). Although pH decreased progressively with increasing fertilizer rate in unplanted and planted substrate, the decline was more pronounced in the planted pots (Supplementary Figure S2A). In the rhizosphere, ammonium uptake by petunia roots led to a drop in pH which was stronger at higher fertilizer rates (100 and 200 mg·L^−1^ N). In contrast, pH reductions in the unplanted substrate likely resulted from microbial nitrification alone. These results underscore the critical role of plant–fertilizer interactions in shaping the rhizosphere chemical environment, where roots not only take up nutrients but also modify substrate pH (Zhang et al., 2021), which indirectly influences nutrient availability.

We contrasted the tissue nutrient concentrations observed in this project with previously recommended ranges suggested by Gibson et al. (2007) and Veazie et al. (2025). Even at the highest rate of 200 mg·L^−1^ N, the P tissue concentrations were below the recommended levels in both cultivars, which is explained by the low phosphorus levels (1.32% P) in our fertilizer (Table 1). At 25 or 100 mg·L^−1^ N, the K tissue concentrations were below recommended range in Picobella. Accordingly, the K concentrations in the substrate fertilized with 25 mg·L^−1^ N was the same as 100 mg·L^−1^ N, and both were lower than at 200 mg·L^−1^ N (Table 1). All together this suggests that only the fertilizer rate of 200 mg·L^−1^ N appeared to exceed the plant K requirement, leading to K accumulation in the substrate. Although Ca, Mg, S, B, and Zn concentrations were within the recommended ranges, Fe and Mn exceeded the upper limits by up to fivefold and threefold, respectively (Gibson et al., 2007; Veazie et al., 2025). These findings align with the increased Fe and Mn concentration in the substrate driven by pH acidification. This suggests that petunia growth and health responses to fertilizer rate were influenced by the amount of nutrients applied and the effects of nutrient uptake on substrate chemical properties. Specifically, ammonium-N uptake, which at high rates increased micronutrient availability by reducing substrate pH.

### 4.2 Peat-based substrate serves as microbial reservoir for petunia root bacteriome assembly

Peat-based substrate is often viewed as unsuitable to supporting beneficial microorganisms, and its microbiome as not favorable for disease suppression (Hoitink and Boehm, 1999; Krause et al., 2001; Pot et al., 2022). Old peat is more decomposed and may not provide an adequate energy source for non-indigenous microbial inoculants (Van Gerrewey et al., 2020a). Moreover, the native microbial communities of young peat are reported to limit the permanent establishment of microbial inoculants (Van Gerrewey et al., 2020b). In contrast, compost has been shown to improve the efficacy of beneficial microorganisms (Krause et al., 2001). Accordingly, the microbiome of peat-based substrates is contrastingly different than the microbiome of composts (Pot et al., 2022). Sphagnum peat is less diverse than coir, leaf compost, and pine bark, but more diverse than mineral substrates (i.e., rockwool) (Grunert et al., 2016; Montagne et al., 2017; Valles-Ramirez et al., 2023).

The bacteriome of soilless substrates is shaped by both substrate materials and their manufacturing processes (e.g., liming) (Montagne et al., 2017; Pot et al., 2022). Each substrate component harbors unique microbial communities that collectively influence the final microbial structure of the substrate mix (Montagne et al., 2017; Pot et al., 2022; Valles-Ramirez et al., 2023). For instance, the bacteriome of raw sphagnum peat moss is dominated by phylum Actinomycetota (>80%), consistent with its naturally low pH (Montagne et al., 2017; Valles-Ramirez et al., 2023). In contrast, the bacteriome of a commercial peat-based substrate used to grow *Impatiens × walleriana* was dominated by Pseudomonadota (formerly Proteobacteria), Actinomycetota, and Bacteroidota (Quijia Pillajo et al., 2024). Similarly, our substrate mix was dominated by phyla Pseudomonadota, Actinomycetota, and Acidobacteriota. The shift from an Actinomycetota-dominated community in raw peat to a Pseudomonadota-dominated community in horticultural substrate mixes is linked to pH changes (Montagne et al., 2017; Valles-Ramirez et al., 2023), likely introduced during substrate manufacturing. At finer taxonomic resolution, our substrate mix was dominated by genera such as *Burkholderia-Caballeronia-Paraburkholderia*, *Acidothermus*, *Mucilaginibacter*, and *Mycobacterium*, which contrasts with the microbial communities reported in other soilless substrates (Anzalone et al., 2022; Valles-Ramirez et al., 2023; Quijia Pillajo et al., 2024). For example, a peat-based substrate used in impatiens culture was dominated by *Streptomyces, Actinocatenispora, Chitinophaga*, and *Flavobacterium* (Quijia Pillajo et al., 2024). The differences between our substrate, raw sphagnum peat, and other commercial peat-based substrates highlight material selection and origin, and processing steps as key drivers shaping the substrate microbial communities, and the potential to use them as tools for substrate engineering to improve plant performance through beneficial plant–microbe interactions.

The two-step model proposes that microbes are first recruited from the soil to the rhizosphere through root exudation and subsequently filtered into the endosphere by plant host genetics (Bulgarelli et al., 2013). Thus, a reduction in alpha diversity in the rhizosphere and endosphere fractions is expected (Edwards et al., 2015), and has been shown in several species like tomato (Barajas et al., 2020). However, exceptions have been reported when comparing ruderal plants versus commercial cultivars. For instance, ruderal plants were shown to harbor a larger bacterial diversity than a commercial tomato cultivar and the soil used to grow them (Barajas et al., 2020). The reported observation is associated with genetic loss for traits that facilitate the establishment of microorganism in the root compartments (rhizosphere and endosphere) of crop species (Pérez-Jaramillo et al., 2018; Barajas et al., 2020). Similar to food crop species, the petunia rhizosphere alpha diversity was lower than the substrate alpha diversity (unplanted pots), and the endosphere alpha diversity was lower than both the rhizosphere and unplanted substrate alpha diversities. Our results indicate that petunias selectively recruit microbiomes from the substrate to the rhizosphere and endosphere. Beta diversity and community composition analysis supported our conclusions as all sample types (bulk, unplanted, rhizosphere, and endosphere) clustered separately. Another hallmark in the root microbiome of wild species is the high relative abundance of Bacteroidota, while Pseudomonadota and Actinomycetota are more abundant in modern crop species (Pérez-Jaramillo et al., 2018). In agreement, the rhizosphere and endosphere bacterial communities of the petunia cultivars analyzed here were dominated by phyla Pseudomonadota and Actinomycetota, while Acidobacteriota and Bacteroidota ranked 3rd and 4th, respectively (Figures 5E, 8E). The endosphere bacteriome of petunia line V26 grown in soil was similarly shown to be dominated by Pseudomonadota, Actinomycetota, and Bacteroidota, but not Acidobacteriota (Bodenhausen et al., 2019). Further, evaluations comparing wild petunias (i.e., *Petunia axillaris* and *Petunia integrifolia*) and a larger set of commercial cultivars would allow better evaluation of the impact of plant domestication in the petunia microbiome. Only 13 genera in the rhizosphere and endosphere were not present in the unplanted samples, suggesting that the substrate and irrigation water are the main contributor of microbial diversity for microbiome assembly. Although to a lesser extent than soil, the seed microbiome can also contribute to the endosphere microbiome (Beirinckx et al., 2020). The five genera found exclusively in the endosphere may have originated from the petunia seed microbiome.

In comparison to alternative soilless substrates, peat-based ones have a reduced presence of beneficial bacterial genera like *Bacillus*, *Burkholderia*, *Paenibacillus*, *Pseudomonas*, *Serratia*, and *Streptomyces* (Pot et al., 2022). Various genera linked to plant growth promotion were present in our peat-based substrate, rhizosphere, or endosphere samples (i.e., *Bacillus*, *Leifsonia*, *Paenibacillus*, *Pseudomonas*, *Streptomyces*, *Chitinophaga*, *Chryseobacterium*, *Flavobacterium*, *Sphingomonas*, and *Stenotrophomonas*) (Radzki et al., 2013; Berg and Martinez, 2015; Chauhan et al., 2015; Carrión et al., 2019; Luo et al., 2019; Nordstedt et al., 2020; Nordstedt et al., 2021; Cui et al., 2022; Afzal et al., 2023). However, among the genera in the petunia rhizosphere core microbiome, only *Nocardia, Mycobacterium, Mucilaginibacter*, *Burkholderia-Caballeronia-Paraburkholderia, Devosia*, and *Bradyrhizobium* have been reported to promote plant growth (Egamberdiyeva, 2007; South et al., 2021; Fan and Smith, 2022; Lamrabet et al., 2024; Zhang et al., 2024). When considering only rhizosphere samples, 29 core genera were identified. However, when both rhizosphere and endosphere samples were considered, only *Nocardia*, *Mycobacterium*, *Mucilaginibacter*, and *Legionella* were present in both compartments.

### 4.3 Fertilizer rate indirectly influences bacterial diversity in the rhizosphere by reducing the pH

Substrate components, irrigation method, phenological stage, and fertilization method influence the microbiomes in soilless culture systems (Montagne et al., 2017; Li et al., 2022; Valles-Ramirez et al., 2023; Quijia Pillajo et al., 2024). There was a contrasting response to fertilizer rate in the unplanted substrate, rhizosphere, and endosphere bacterial diversity. Unplanted substrate had higher alpha diversity than bulk substrate (i.e., out of the bag). However, additional taxa observed in unplanted substrate might result from additional bacterial taxa introduced by the irrigation water. Fresh water applied via greenhouse irrigation is a source of microbial diversity influenced by factors such as origin (rain vs. ground) and storage management (Picot et al., 2020). In our experimental set-up, all fertilizer treatments were made with non-sterile distilled water. Fertilizer rate did not have an effect on bacterial diversity in the unplanted substrate. In contrast, N-fertilization can reduce soil bacterial diversity (Dai et al., 2018). For instance, long-term N-fertilization alone (urea) reduces bacterial richness and Shannon diversity in grassland soil (Li et al., 2019). However, when N-fertilization is combined with P or K (NPK, NP or NK), it can increase bacterial diversity (Dai et al., 2018).

At the rhizosphere, the microbiome structure and function is not only influenced by nutrient availability, but also by the plant response to nutrient availability (Finkel et al., 2019; Grunert et al., 2019). Nutrient availability modulates root exudate profiles that in turn impact the microbiome structure and function (Pantigoso et al., 2022). In contrast to unplanted substrate, increasing fertilizer rate led to a decrease in alpha diversity in the rhizosphere of both petunia cultivars. Similarly, increasing N fertilization reduced bacteria diversity in rice and peanut rhizosphere (Dong et al., 2021; Zhang et al., 2022). It has been found that a decrease in bacterial diversity can be associated with changes in pH caused by inorganic fertilization (Zhang et al., 2022). Accordingly, in this current experiment, the increase in fertilizer rate was accompanied by a decline in substrate pH. The N-form (NH_4_^+^, ammonium and NO_3_^−^, nitrate) has a large influence on substrate pH, because ammonium-based fertilizers lead to substrate acidification (Argo and Fisher, 2003). Ammonium fertilization leads to acidification due to the accumulation of H^+^ produced during microbial nitrification and ammonium uptake by roots (Havlin et al., 2014). The observed pH drop in planted substrate was expected since the 20N–1.3P–15.8K petunia FeED fertilizer used in this experiment has a high percentage of NH_4_^+^ (~40%) and is commonly used to acidify the substrates (Argo and Fisher, 2003). Bacterial diversity at the rhizosphere is shaped by the combined effects of nitrogen form and root influence, primarily due to soil pH changes associated with root uptake of NH_4_^+^ and NO_3_^−^ (Zhang et al., 2021). Both shifts toward more acidic or alkaline pH lead to reduced alpha diversity (Xiong et al., 2024). Zhang et al. (2021) showed that while NH_4_^+^ fertilization decreased the pH (from ~5.5 to ~4.7) and NO_3_^−^ fertilization increased it (from ~5.5 to ~6.2), both lead to a decrease in alpha diversity of the maize rhizosphere. Since fertilization rate did not affect alpha diversity in our unplanted substrate, it seems that the observed decrease in bacterial diversity in the petunia rhizosphere is an indirect effect of fertilization rate on the substrate pH.

### 4.4 Fertilizer rate shows divergent responses in the bacterial community composition in unplanted substrate and the rhizosphere

In soil, inorganic fertilization increases the relative abundance of copiotrophic bacteria (i.e., Pseudomonadota, Actinomycetota, and Bacteroidota), but decreases oligotrophic bacteria (i.e., Acidobacteria, Planctomycetes, and Verrucomicrobia) (Pan et al., 2014; Dai et al., 2018; Li et al., 2019). For instance, in grassland soil both N and K fertilization increases the abundance of Actinomycetota, but reduces the abundance of Firmicutes and Verrucomicrobia (Pan et al., 2014). In our unplanted substrate, increasing the fertilization rate increased the relative abundance of Pseudomonadota, and decreased the relative abundance of Myxococcota and Bacteroidota. Furthermore, LEfSe analysis identified Pseudomonadota and Actinomycetota as biomarkers of the 200 mg·L^−1^ N treatment, highlighting their copiotrophic lifestyle (Pan et al., 2014; Dai et al., 2018; Li et al., 2019). Consistent with the copiotrophic lifestyle of Pseudomonadota, most of the differentially abundant genera within this phylum were enriched (16 out of 20) at higher fertilizer rates. Other genera also enriched at high fertilizer rates and belonging to copiotrophic phyla were *Mycobacterium* and *Nocardioides* (both Actinomycetota) and *TM7a* (Patescibacteria). In contrast and consistent with the oligotrophic lifestyle of Planctomycetota (Li et al., 2019), *Fimbriiglobus*, *Planctopirus*, and *Schlesneria*, all members of this phylum, were reduced at higher fertilization rates.

However, rhizosphere and unplanted substrate bacterial communities responded differently to fertilization. Despite the copiotrophic response of Pseudomonadota in the unplanted substrate (Figure 3D) and its identification as a biomarker in the rhizosphere of petunias fertilized with 200 mg·L^−1^ N (Supplementary Figure S5), the relative abundance of Pseudomonadota in the rhizosphere was not different across fertilization rates. In contrast to the unplanted substrate, only a small proportion (9 out of 33) of differentially abundant genera within the phylum Pseudomonadota in the rhizosphere were enriched at higher fertilizer rate (Figure 6). *Burkholderia-Caballeronia-Paraburkholderia* was the dominant genera within phylum Pseudomonadota in the rhizosphere (Figure 2E) and is known to tolerate acid environments (Pongmala et al., 2022; Csorba et al., 2024; Zhou et al., 2024). The *Burkholderia-Caballeronia-Paraburkholderia* increase in the rhizosphere and the unplanted substrate might result from their low pH-tolerance advantage. In agreement with our results, NH4^+^ fertilization has been shown to increase order Burkholderiales and genus *Burkholderia* in the rhizosphere (Zhang et al., 2021). In contrast to chemical fertilization, manure application increases nutrient availability with no soil acidification, but it decreases the abundance of family Burkholderiaceae (Zhang et al., 2022). Among the bacteria genera enriched in response to increasing fertilizer rate, *Dyella, Methylovirgula*, and *Rhodanobacter* tolerate low pH environments, but *Bosea* and *Luteimonas* are common to neutral and high pH, respectively (Carlson et al., 2019; Zhou et al., 2024). Similarly, the genera that decreased at higher fertilizer rate also showed diverse pH preferences, suggesting that fertilization effects are not solely governed by pH. For instance, *Bauldia, Dokdonella, Inquilinus, Nevskia, Reyranella, Rhodovastum, Roseiarcus*, and *SWB02* tolerate low pH environments (pH < 5.5). *Ellin 6067* and *IS-44* live in neutral environments (pH 5.5–7.5), and *Ramlibacter*, *Rhodopseudomonas,* and *Sphingomonas* inhabit high pH environments (pH > 7.5) (Zhou et al., 2024). Notably, in another study, *Sphingomonas* responded differently with increased abundance in response to NO_3_^−^ fertilization, which was accompanied by a rise in soil pH (Xiong et al., 2024). Our results and the findings of Zhang et al. (2021) support the idea that fertilization alters bacterial community composition by both directly increasing nutrient availability and indirectly modulating soil or substrate pH.

Actinomycetota are generally considered copiotrophs, adapted to high-nutrient environments (Fierer et al., 2012; Leff et al., 2015). Accordingly, all differentially abundant genera within the Actinomycetota phylum, identified in the unplanted substrate and the rhizosphere increased in response to fertilization rate. In addition, they were positively correlated with the amount of water-soluble nutrients but negatively correlated to pH. Armatimonadota, Bacteroidota, Myxococcota, Planctomycetota, and Verrucomicrobiota are oligotrophs (i.e., adapted to low nutrient environments) (Li et al., 2019). Accordingly, all differentially abundant genera within oligotrophic phyla, identified in the unplanted substrate and the rhizosphere decreased in response to fertilization rate. Moreover, they were negatively correlated with nutrient concentrations in the substrate and positively correlated to pH. As previously reported, fertilization generally favors copiotrophic bacteria including some Pseudomonadota, and it reduces the abundance of oligotrophic bacteria like *Verrucomicrobiota*. However, it is important to note that taxonomic groups can include both more-oligotrophic and more-copiotrophic members (Stone et al., 2023; Dragone et al., 2024). These findings suggest that bacterial life history strategies (i.e., copiotrophic vs. oligotrophic), in conjunction with pH tolerance, play an important role in shaping bacterial community composition in response to fertilization.

### 4.5 Fertilizer rate does not influence alpha diversity, but it affects community composition in the endosphere

Fertilizer rate did not influence bacterial diversity in the endosphere of both petunia cultivars. Similarly, urea application did not affect the endophyte diversity of sorghum (Li et al., 2024). Mineral and organic fertilization also did not affect the fungal and bacterial diversity in sorghum roots endosphere (Sun et al., 2021). Compared to the rhizosphere, beta diversity analysis based on Bray–Curtis dissimilarity showed a less pronounced effect of fertilizer rate, cultivar, and their interaction on the root bacterial community composition. Despite the significant effect of fertilizer rate on beta diversity, our analysis (Kruskal-Walli’s test and LEfSe) did not find differentially abundant taxa or biomarkers associated with increasing fertilization. Pseudomonadota and Actinomycetota accounted for about 75% of the endosphere bacterial community. While the average relative abundance of phylum Actinomycetota increased in Wave, it decreased in Picobella. In agreement, N-fertilization (urea) is reported to increase the relative abundance of phylum Actinomycetota and decrease the abundance of phylum Pseudomonadota (Li et al., 2024). Overall, the impact of fertilization on the endosphere was weaker than in the rhizosphere or unplanted substrate. Similarly, Sun et al. (2021) observed limited fertilization effects on the phyllosphere and root endophyte communities of sorghum. These findings support the idea that endosphere communities are primarily shaped by host genetics (Trivedi et al., 2020), whereas changes in substrate properties contribute more to fertilization effects observed in soil/substrate and rhizosphere communities.

## 5 Conclusion

Soilless substrates have low nutrient buffering capacity; thus, root zone management requires regular monitoring of pH and nutrients. Accordingly, fertilizer management has been identified as a key research and educational priority for specialty crop growers using or transitioning to soilless substrates (Fields et al., 2023). In petunias grown in a peat-based substrate, fertilization rate shapes bacterial communities in both the rhizosphere and root endosphere. However, fertilization has a stronger effect in the rhizosphere. We showed that while the impact of fertilization on the bacteriome of unplanted substrate can be attributed to increased nutrient availability, in the rhizosphere it appears to be a result of both the direct increase in nutrient availability and pH changes driven by N uptake, which can also depend on the N-form applied. Substrate pH and fertilization programs are optimized to maximize crop performance. However, both practices also modulate microbial communities in container grown crops and could potentially become tools to advance the use of microbial inoculants to harness substrate microbiomes for enhanced plant growth and improved fertilizer use efficiency. However, to unlock the full potential of plant associated microbiomes, further research is needed to understand other factors (i.e., pesticides, plant growth regulators, and non-microbial biostimulants) influencing both native and introduced microbial communities.

## Data Availability

The raw sequencing data generated in this study have been deposited in the National Center for Biotechnology Information (NCBI) Short-Read Archive (SRA) under the BioProject PRJNA1282715.

## Supplementary material

Supplementary FiLe 1 Environmental variables correlation with PCoA.

Supplementary FiLe 2 Full differential abundance analysis across sampling compartments.

Supplementary FiLe 3 Full differential abundance analysis of unplanted substrate samples.

Supplementary FiLe 4 Full differential abundance analysis of rhizosphere samples.
