# Supplementary material for: Fertilization influences the substrate, rhizosphere, and endosphere bacteriome of Petunia × hybrida
Source: Front Microbiol. 2025 Dec 5;16:1719754. doi: 10.3389/fmicb.2025.1719754 (PMC12714982; doi:10.3389/fmicb.2025.1719754)
Supplement: Supplementary file 5 [file Data_Sheet_1.pdf]

# Fertilization influences the substrate, rhizosphere, and endosphere bacteriome of *Petunia* × *hybrida*

**Juan Quijia-Pillajo[1], Laura J. Chapin[1], James S. Owen Jr.[2], James E. Altland[2], and Michelle L. Jones[1*]**

ORCID ID: 0000-0002-2781-742X (J.Q.P.)
ORCID ID: 0000-0003-4876-5320 (L.J.C)
ORCID ID: 0000-0002-7791-5407 (J.S.O. Jr.)
ORCID ID: 0000-0002-2979-5527 (M.L.J.)

[1]Department of Horticulture and Crop Science, The Ohio State University, Wooster, OH 44691, USA
[2]Application Technology Research Unit, United States Department of Agriculture (USDA)-Agricultural Research Service, Wooster, OH, USA

*** Correspondence:**
Michelle Jones
jones.1968@osu.edu

# *Supplementary Material*

## 1 Supplementary Figures and Tables

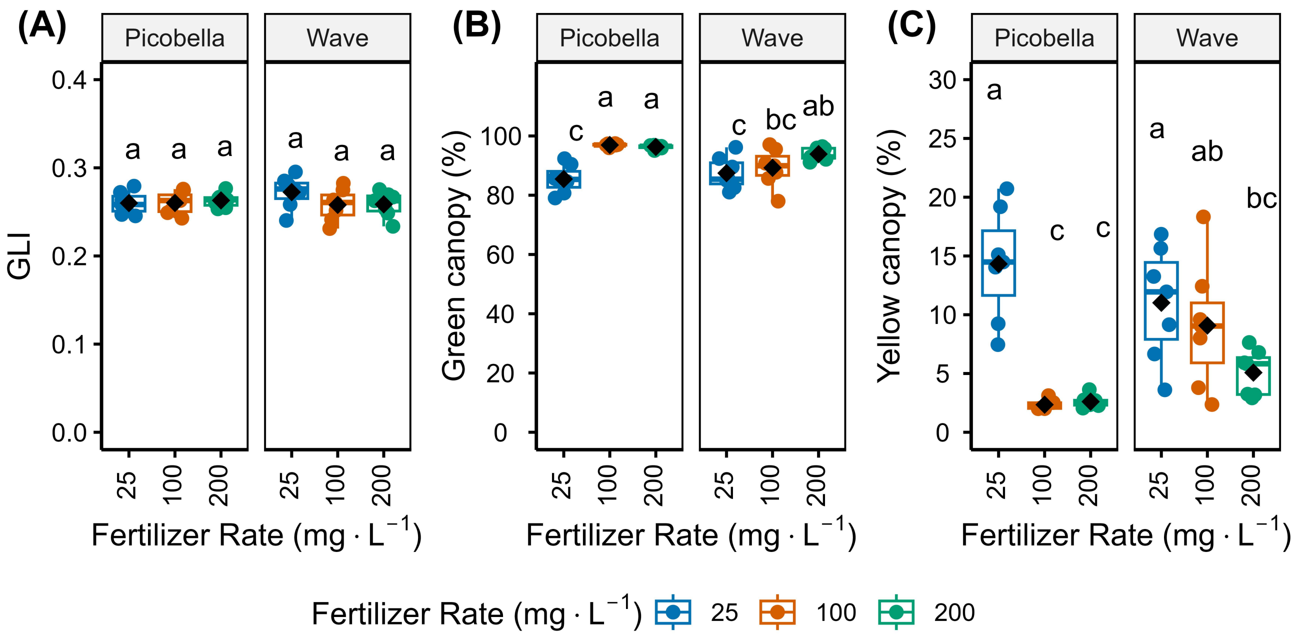


**Supplementary Figure S1**. Effect of fertilizer rate on physiological responses of two petunia cultivars. Petunia 'Picobella Blue' and 'Wave Purple' were grown under three fertilizer rates (25, 100, and 200 mg·L$^{-1}$ N). Fertilizer solutions were prepared with a 20N–1.3P–15.8K Petunia FeED water soluble fertilizer. Boxplots showing the effects of fertilizer rate on green leaf index (GLI) **(A)**, green canopy percentage **(B)**, and yellow canopy percentage **(C)**. Each boxplot displays the median (center line), interquartile range (box), mean (black diamonds) and individual data points (n = 7). Different lowercase letters indicate statistically significant differences based on Tukey's honestly significant difference (HSD) test ($p \leq 0.05$).

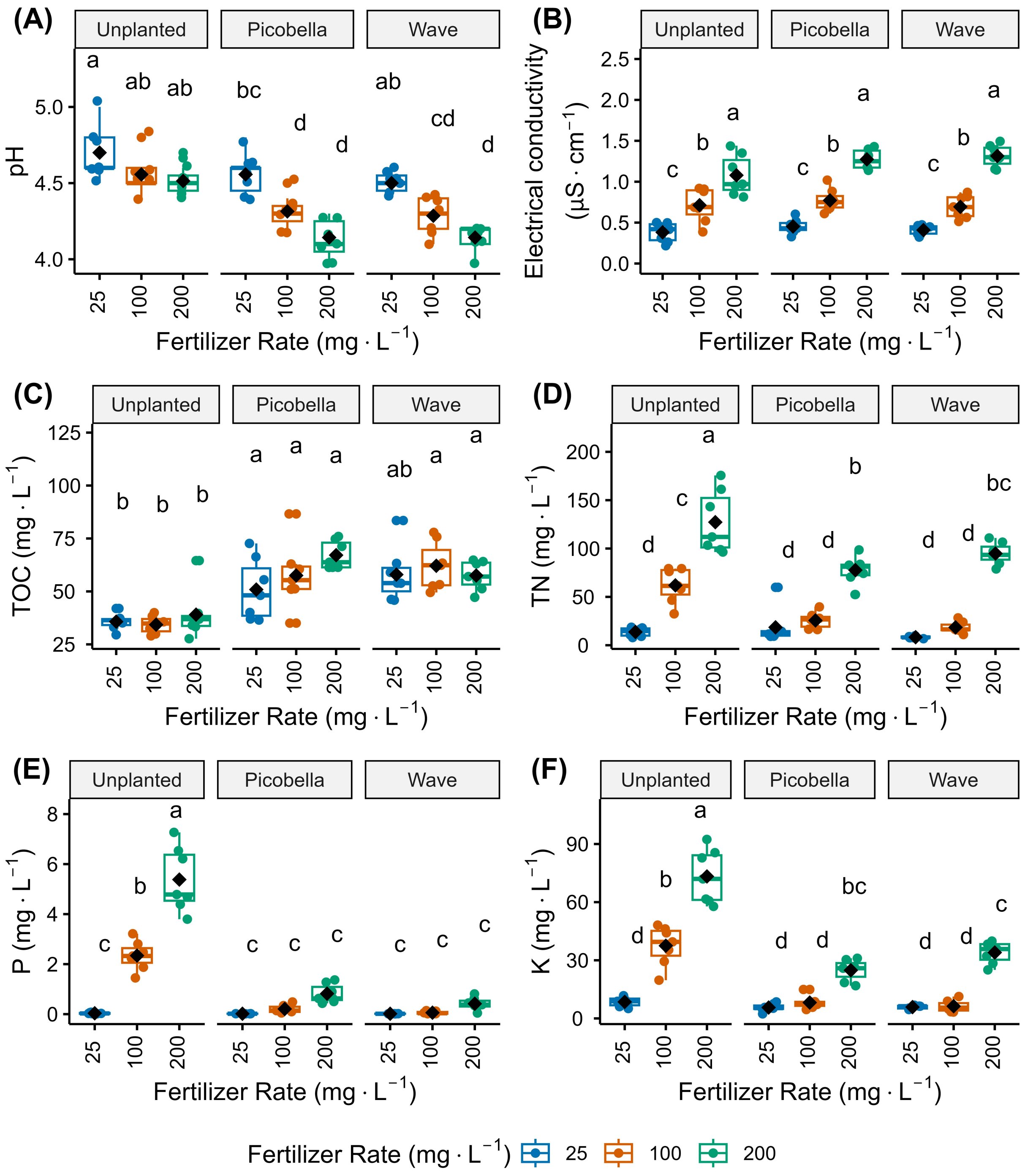


**Supplementary Figure S2.** Effect of fertilizer rate on substrate chemical properties of unplanted and planted substrate. Unplanted substrate and petunia ‘Picobella Blue’ and ‘Wave Purple’ received three fertilizer rates (25, 100, and 200 mg·L$^{-1}$ N). Fertilizer solutions were prepared with a 20N–1.3P–15.8K Petunia FeED water soluble fertilizer. Boxplots showing the effects of fertilizer rate on pH **(A)**, electrical conductivity **(B)**, TOC (Total organic carbon) **(C)**, TN (Total nitrogen) **(D)**, P (Phosphorus) **(E)**, and K (Potassium) **(F)**. Each boxplot displays the median (center line), interquartile range (box), mean (black diamonds) and individual data points (n = 7). Different lowercase letters indicate statistically significant differences based on Tukey's honestly significant difference (HSD) test ($p \leq 0.05$).

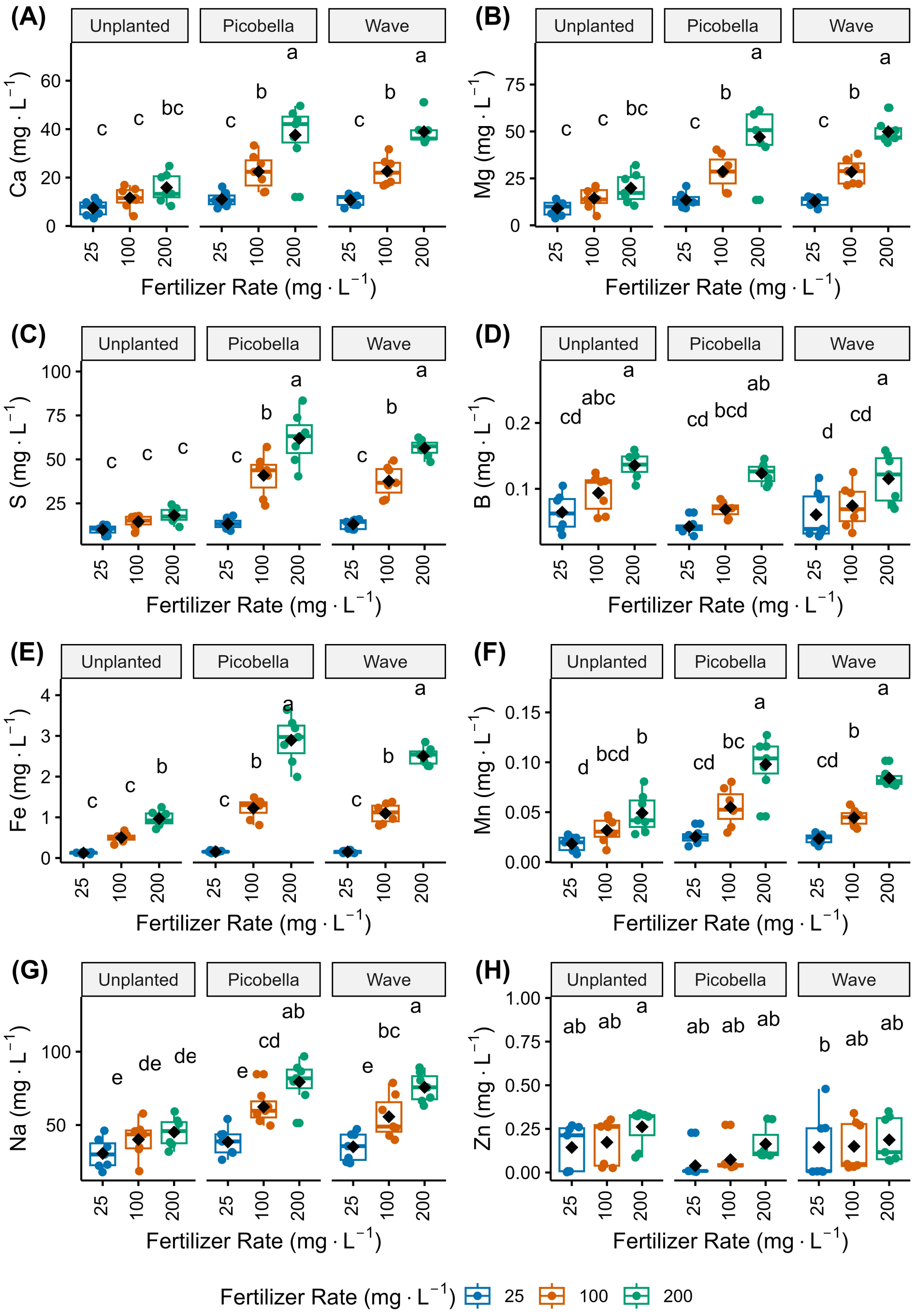


**Supplementary Figure S3.** Effect of fertilizer rate on substrate chemical properties of unplanted and planted substrate. Unplanted substrate and petunia ‘Picobella Blue’ and ‘Wave Purple’ received three fertilizer rates (25, 100, and 200 mg·L$^{-1}$ N). Fertilizer solutions were prepared with a 20N–1.3P–15.8K Petunia FeED water soluble fertilizer. Boxplots showing the effects of fertilizer rate on Ca (Calcium) **(A)**, Mg (Magnesium) **(B)**, S (Sulfur) **(C)**, B (Boron) **(D)**, Fe (Iron) **(E)**, Mn (Manganese) **(F)**, Na (Sodium) **(G)**, and Zn (Zinc) **(H)**. Each boxplot displays the median (center line), interquartile range (box), mean (black diamonds) and individual data points (n = 7). Different lowercase letters indicate statistically significant differences based on Tukey's honestly significant difference (HSD) test ($p \leq 0.05$).

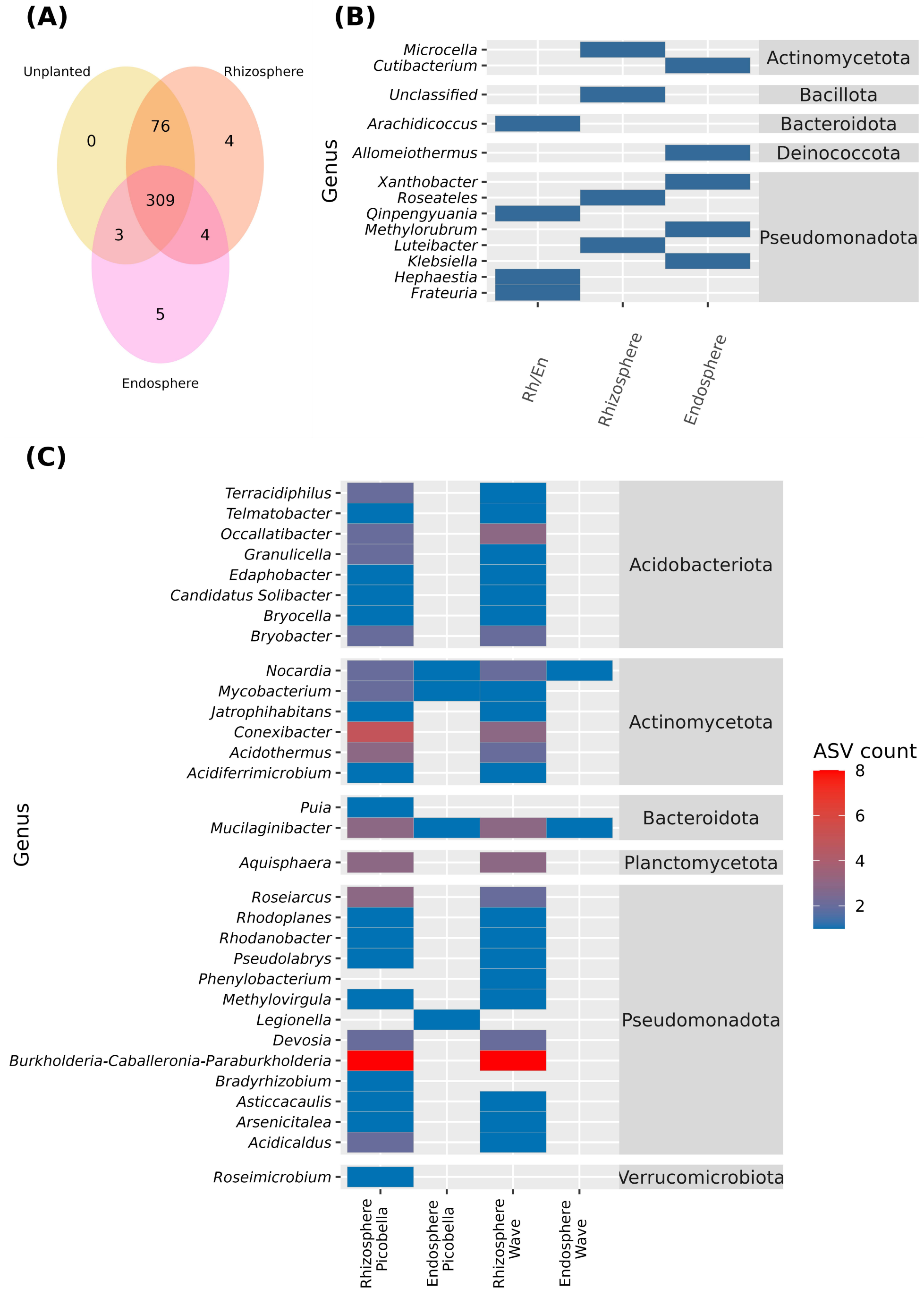


**Supplementary Figure S4.** Bacteriome of unplanted substrate, rhizosphere, and endosphere samples. Rhizosphere and endosphere were collected from Petunia ‘Picobella Blue’ and ‘Wave Purple’ grown under three fertilizer rates (25, 100, and 200 $mg\cdot L^{-1}$ N) for 7 weeks. Unplanted substrate also received three fertilization rates for 7 weeks. Venn diagram showing the number of shared bacterial genera in unplanted substrate, rhizosphere and roots **(A)**. Heatmap displaying presence/absence of genera unique to rhizosphere and roots **(B)**. R/R indicates present in both. Heatmap displaying core bacteriome in rhizosphere and roots of petunia ‘Picobella Blue’ and ‘Wave Purple’ **(C)**. Core microbiome was identified at ASV level, the number of ASVs in each genus is indicated by the tile color.

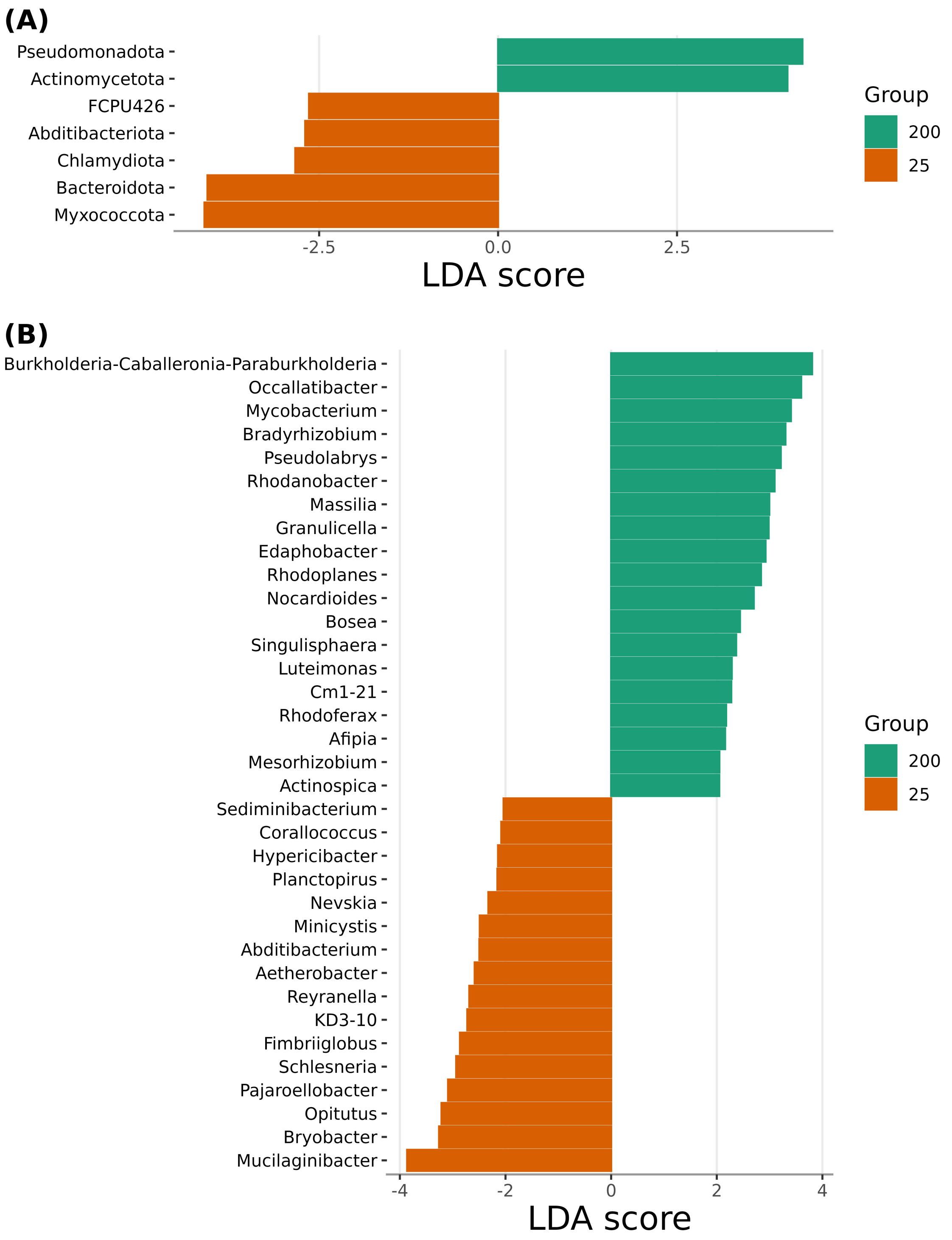


**Supplementary Figure S5.** Linear discriminant analysis (LDA) effect size (LEfSe) results on unplanted substrate bacteriome. Histogram of the LDA scores computed for phyla **(A)** and genera **(B)** differentially abundant between 25 and 200 mg·L$^{-1}$ N. Unplanted substrate received two fertilization rates (25 and 200 mg·L$^{-1}$ N) for 7 weeks.

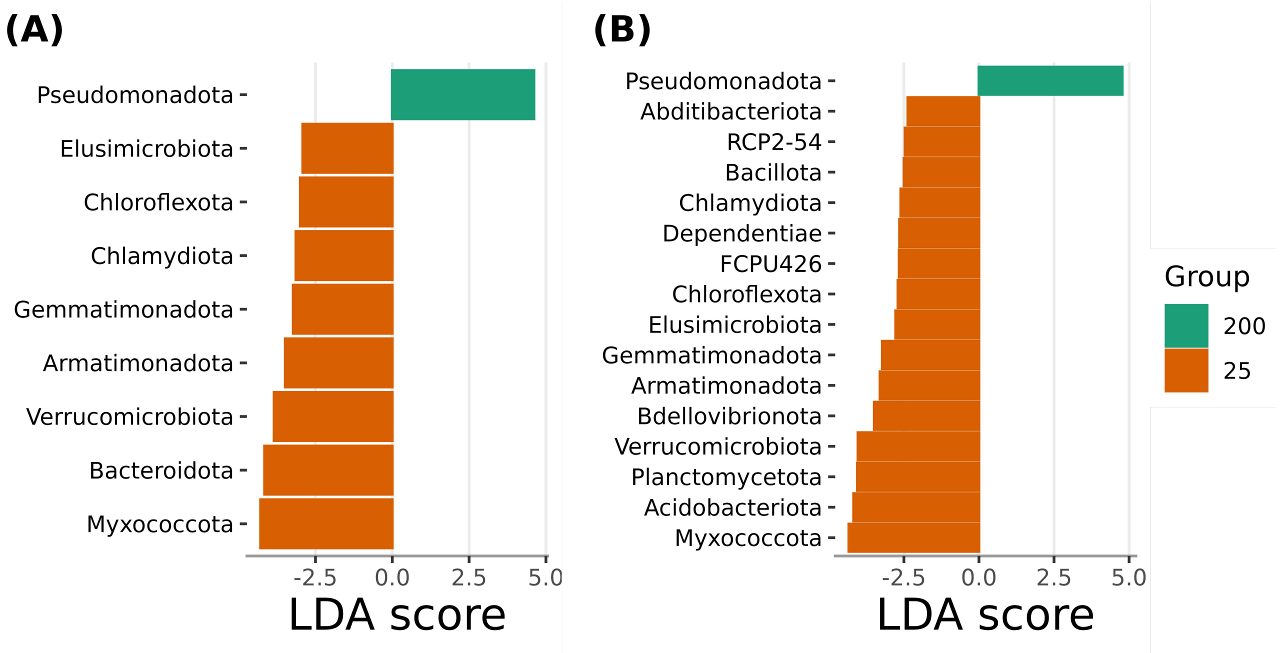


**Supplementary Figure S6.** Linear discriminant analysis (LDA) effect size (LEfSe) results on rhizosphere bacteriome of petunia 'Picobella Blue' **(A)** and 'Wave Purple' **(B)**. Histogram of the LDA scores computed for phylum differentially abundant between 25 and 200 mg·L$^{-1}$ N. Rhizosphere samples were collected from petunias grown under two fertilizer rates (25 and 200 mg·L$^{-1}$ N) for 7 weeks.

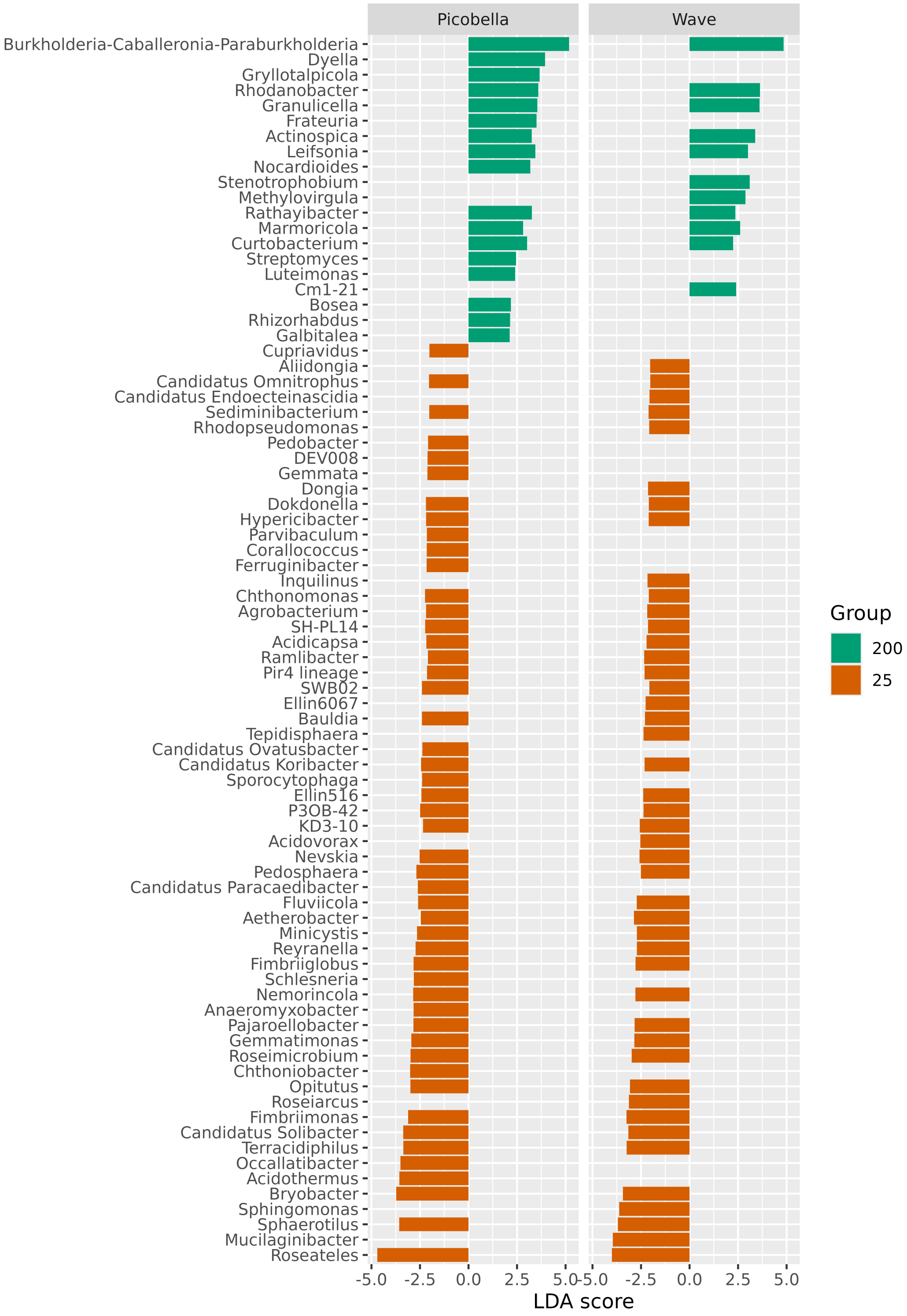


**Supplementary Figure S7.** Linear discriminant analysis (LDA) effect size (LEfSe) results on rhizosphere bacteriome of petunia 'Picobella Blue' (left) and 'Wave Purple' (right). Histogram of the LDA scores computed for genera differentially abundant between 25 and 200 mg·L$^{-1}$ N. Rhizosphere samples were collected from petunias grown under two fertilizer rates (25 and 200 mg·L$^{-1}$ N) for 7 weeks.
